# Microglial priming by IFN‐γ involves STAT1‐mediated activation of the NLRP3 inflammasome

**DOI:** 10.1111/cns.70061

**Published:** 2024-10-11

**Authors:** Haili He, Xiaomei Zhang, Hui He, Gaojie Xu, Liangyuan Li, Chengyan Yang, Yu‐e Liu, Zili You, Jinqiang Zhang

**Affiliations:** ^1^ Resource Institute for Chinese and Ethnic Materia Medica, Guizhou University of Traditional Chinese Medicine Guiyang China; ^2^ School of Life Science and Technology, Center for Informational Biology University of Electronic Science and Technology of China Chengdu China

**Keywords:** interferon‐gamma, lipopolysaccharide, microglial priming, NLRP3 inflammasome, STAT1

## Abstract

**Background:**

Inflammatory and immune responses in the brain that contribute to various neuropsychiatric disorders may begin as microglial “priming”. Interferon (IFN)‐γ is known to cause microglial priming, but the mechanism is unclear.

**Methods:**

We examined the effects of IFN‐γ on gene expression, microglial activation, inflammatory and immune responses and activity of the NLRP3 inflammasome in primary microglia and in the brains of mice.

**Results:**

Our results showed that treating microglial cultures with IFN‐γ induced a hedgehog‐like morphology and upregulated markers of microglial activation (CD86, CD11b) and pro‐inflammatory molecules (IL‐1β, IL‐6, TNF‐α, iNOS), while downregulating markers of microglial homeostasis (CX3CR1, CD200R1), anti‐inflammatory molecules (MCR1, Arg‐1) and neurotrophic factors (IGF‐1, BDNF). IFN‐γ also upregulated markers of NLRP3 inflammasome activation (NLRP3, caspase‐1, gasdermin D, IL‐18). This particular transcriptional profiling makes IFN‐γ‐primed microglia with exaggerated responses upon lipopolysaccharide (LPS) stimulation. The level of NLRP3, caspase‐1, gasdermin D, IL‐1β, IL‐18, TNF‐α and iNOS in microglia cultures treated with both IFN‐γ and LPS were highest than with either one alone. Injecting IFN‐γ into the lateral ventricle of mice induced similar morphological and functional changes in hippocampal microglia as in primary microglial cultures. The effects of IFN‐γ on NLRP3 inflammasome and microglia from cultures or hippocampus were abolished when STAT1 was inhibited using fludarabin. Injecting mice with IFN‐γ alone or together with LPS induced anxiety‐ and depression‐like behaviors and impaired hippocampus‐dependent spatial memory; these effects were mitigated by fludarabin.

**Conclusions:**

IFN‐γ primes microglia by activating STAT1, which upregulates genes that activate the NLRP3 inflammasome. Inhibiting the IFN‐γ/STAT1 axis may be a way to treat neurodegenerative diseases and psychiatric disorders that involve microglial priming.

## Graphical abstract

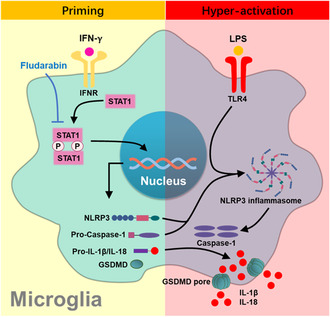

## 1 INTRODUCTION

Microglia are the resident immune cells of the central nervous system,^1^ where they monitor for the presence of pathogens and mount immune responses accordingly, while also phagocytosing apoptotic cells and promoting neurogenesis.^2^, ^3^ Microglia are not only passive defenders of the brain: they also help modify and maintain neuronal circuitry underlying high‐level activities such as cognition and emotion.^4^, ^5^ The same microglial functions that are crucial for maintaining brain function can, if hyperactivated, lead to pathology. For example, stimulating microglia with LPS causes them to take on an ameboid shape^6^ with shortened branches, which normally help them monitor their surroundings.^7^ Ameboid microglia begin secreting abundant pro‐inflammatory cytokines such as interleukin (IL)‐1β and tumor necrosis factor (TNF)‐α,^8^ which can lead to neuroinflammation.^9^ If microglia are repeatedly activated in this way, the resulting chronic neuroinflammation may increase the risk of neurodegenerative disease,^10^ depression‐ or anxiety‐like behaviors,^11^ or cognitive decline.^12^, ^13^ Preventing excessive microglial activation is therefore important for protecting neural circuits.

One challenge to preventing microglial hyperactivation is that an initial inflammatory stimulus can “prime” microglia to respond even more strongly to subsequent stimuli,^14^, ^15^, ^16^ and such priming is thought to contribute to systemic inflammation and neurodegeneration, especially with aging.^17^ A key driver of microglial priming appears to be interferon (IFN)‐γ,^12^ which is produced primarily by activated T cells and which promotes inflammation and antiviral immunity.^18^, ^19^ Indeed, IFN‐γ has often been used in the laboratory to boost microglial responses to LPS or amyloid‐β peptide.^20^ Elevated levels of IFN‐γ have been found in the serum or brain of individuals with depression,^21^ autism spectrum disorder,^22^ schizophrenia^23^, ^24^ or Alzheimer's disease.^25^ Treating cancer patients with IFN‐γ increases risk of depression.^26^ Conversely, knocking out IFN‐γ from mice promotes hippocampal plasticity and cognitive performance,^27^ and knocking it out from mice mitigates symptoms of Alzheimer's disease, Parkinson's disease and autoimmune encephalomyelitis.^28^

Many studies have demonstrated that NLRP3 inflammasome activation plays a major role in microglial priming, thereby promoting an exaggerated microglial response to later immune challenges.^29^, ^30^, ^31^ Thereby, we hypothesized that IFN‐γ may induce microglial priming through STAT1‐mediated the NLRP3 inflammasome activation. Here we explored how IFN‐γ primes microglia in an effort to identify potential druggable targets in neuropathologies involving microglial hyperactivation. We performed our experiments with primary cultures of microglia and with mice that were injected with IFN‐γ directly in the brain. Our results identify STAT1‐mediated activation of the NLRP3 inflammasome as at least one pathway through which IFN‐γ primes microglia, which may aid the development of neuroprotective drugs.

## 2 MATERIALS AND METHODS

### 2.1 Primary cultures of microglia and treatments

Primary microglia were isolated from the brains of neonatal C57BL/6J mice (P0–P3) as described^32^ and cultured at 37°C in DMEM/F12 (Gibco, Grand Island, NY, USA) containing 10% fetal bovine serum (Gibco) in an atmosphere of 5% CO_2_. Only cultures containing >98% microglia based on the immunostaining against Iba1 were used in experiments.

Microglia were seeded into 24‐well plates (5 × 10^4^ cells/cm^2^) in DMEM/F12 containing 10% fetal bovine serum and incubated at 37°C for 90 min. Then they underwent one of the following four treatments.

In the first type of experiment, microglia were treated for 24 h with 50 ng/mL recombinant mouse IFN‐γ (PeproTech, Cranbury, NJ, USA) or phosphate‐buffered saline (PBS) as a control. Their morphology was observed under a light microscope (Olympus IX73, Tokyo, Japan).

In a second type of experiment, microglia were treated for 2, 6, 12, and 24 h with 50 ng/mL IFN‐γ alone, 50 ng/mL LPS (Sigma‐Aldrich, St Louis, MO, USA) alone, or both molecules together. Control cultures were incubated with PBS. At each time point, microglial morphology was observed under a light microscope to determine that the morphological differences of microglia appeared in a time‐dependent manner. To examine the transcript levels of molecules involved in microglial NLRP3 inflammasome activation under different treatment conditions, we examined the levels of mRNAs encoding NLRP3, Caspase 1, GSDMD, IL‐1β, IL‐18, TNF‐α and iNOS in microglia treated with PBS, IFN‐γ, LPS or IFN‐γ plus LPS for 24 h.

In a third type of experiment, microglia were treated with IFN‐γ, LPS or IFN‐γ plus LPS in the medium contained with 10 ng/mL ATP (Sigma‐Aldrich), and cultures were incubated another 24 h. The ATP served to promotes the assembly of NRLP3 inflammasome to process interleukin‐1β and 18, and the cultures were analyzed to identify molecules at protein levels that might mediate NRLP3 inflammasome activation.

In a fourth type of experiment, microglia were pretreated for 1 h with 200 μM fludarabin (MedChemExpress, Monmouth Junction, NJ, USA) dissolved in dimethyl sulfoxide (DMSO),^33^ then exposed for 24 h with 50 ng/mL IFN‐γ alone or together with 50 ng/mL LPS. In parallel, microglia were pretreated for 1 h with DMSO vehicle, then exposed for 24 h to the same treatments as in the second type of experiment. These cultures were analyzed to identify molecules involved in activation of the NLRP3 inflammasome and to clarify the role of STAT1 in such activation.

### 2.2 Co‐culture of primary microglia and neural stem/precursor cells

Neural stem/precursor cells (NSPCs) were obtained from the hippocampus of 8‐week‐old male C57BL/6J mice as described^34^ and cultured in the lower chamber of a 24‐well transwell plate (Corning, NY, USA) in DMEM/F12 + GlutaMAX™ (Gibco, Grand Island, NY, USA). Microglia that had been treated for 24 h as described for the “second type of experiment” in Section 2.1 were transferred to the upper chamber of the transwell plate. The plates were incubated for 24 h, after which apoptosis was assessed in NSPCs as described^35^; or for 1 week, after which differentiation of NSPCs was evaluated as described.^35^ During the 1‐week incubation, microglia in the upper chamber were replaced every 24 h.

### 2.3 Mixed cultures of microglia, neurons, astrocytes and oligodendrocytes

NSPCs were cultured in differentiation medium for 1 week as described^35^ to differentiate them into neurons (~ 20%), astrocytes (~ 60%) and oligodendrocytes (~ 20%) to form the mixed cells monolayer. Primary microglia were seed onto the mixed cells monolayer for 24 h to form the mixed culture containing microglia (~ 15%), neurons (~ 18%), astrocytes (~ 52%) and oligodendrocytes (~ 15%). The mixed cultures treated as in the “fourth type of experiment” in Section 2.1.

### 2.4 Phagocytic activity of primary microglia

Microglia were seeded into 24‐well plates (2 × 10^4^ cells/cm^2^) in DMEM/F12 containing 10% fetal bovine serum and incubated for 90 min at 37°C. Then 1 × 10^6^ microbeads of diameter 0.6 μm (Bio‐Rad, Hercules, California, USA) were added to each well, and cultures were incubated another 2 h. The cells were washed with PBS for 15 min, fixed with 4% paraformaldehyde for 1 h, then stained with goat anti‐Iba1 antibody (Iba1; 1:400 dilution; Wako, Japan). Internalization of microbeads was quantified using a fluorescence microscope (model IX73, Olympus, Tokyo, Japan) and ImageJ 1.45J (National Institutes of Health, Bethesda, MD, USA).

### 2.5 In silico analysis of the effects of IFN‐γ on gene expression in microglia

We prepared primary cultures of microglia from the brains of healthy mice, exposed them to IFN‐γ or PBS for 24 h, then harvested RNA from the two samples and compared the expression of numerous genes using RNA sequencing as described.^36^ All sequences were uploaded to the NCBI Sequence Read Archive (submission SUB10788230, BioProject PRJNA787892). Enrichment of differentially expressed genes (fold change ≥2; false discovery rate [FDR] <0.05) in Gene Ontology categories was analyzed using Goatools, while enrichment in Kyoto Encyclopedia of Genes and Genomes pathways was analyzed using Kobas. Potential interactions among proteins encoded by differentially expressed genes were mapped using the Majorbio platform (cloud.majorbio.com).

### 2.6 Experiments in mice

Male 8‐week‐old C57BL/6 mice were purchased from Chengdu Dashuo Laboratory Animals (Chengdu, China) and housed under a standard 12‐h light‐to‐dark cycle in a temperature‐ and humidity‐controlled room. All experiments were approved by the Institutional Animal Care and Use Committee of the Guizhou University of Traditional Chinese Medicine (20231215001).

Animals were randomized to receive single injections of one of the following into the cerebral ventricle (Bregma, −1.0 mm, Lateral, ±1.2 mm; Ventral, 2.0 mm) of 1 μL of PBS, 1 μL of 100 μg/mL IFN‐γ in PBS, 1 μL of 100 μg/mL LPS in saline, or the combination of 0.5 μL of 200 μg/mL IFN‐γ and 0.5 μL of 200 μg/mL LPS. Injections were delivered over a 5‐min period using an automatic microinjection pump with 0.50‐mm needle (RWD Life Science Co., Ltd., Shenzhen, China). The four groups of injected animals were anesthetized with isoflurane (catalog no. R510‐22‐10, RWD Life Science Co., Ltd.) using a tabletop apparatus (catalog no. R640, RWD Life Science Co., Ltd.) and secured in a stereotaxic device (RWD Life Science Co., Ltd.). Animals were sutured and placed on a heating pad for recovery for 2 h until their mobility is restored. A group of control mice was handled in parallel with these four groups but was not injected with anything. Half of the IFN‐γ alone or together with LPS treatment groups of mice were injected intraperitoneally with 10 mg/kg fludarabin 3 days before i.c.v., while half of the other groups of mice were injected intraperitoneally with an equal volume of DMSO.

In this way, we compared the following seven groups in our analysis: Control, PBS, IFN‐γ, LPS, IFN‐γ + LPS, IFN‐γ + fludarabin and IFN‐γ + LPS + fludarabin. These groups were allowed to recover in their cages for 2 days before the experiments described below.

### 2.7 Behavioral analysis of treated mice

Mice were assessed in four behavioral tests. The open field test, which assesses locomotor function and anxiety‐like behavior,^37^ was conducted by placing each mouse in an acrylic plastic box (50 × 50 × 30 cm), and locomotor activity and travel distance were measured during a 5‐min period using OFT 100 software (Techman Tech, Chengdu, China). The tail suspension test, which assesses depression‐like behavior,^38^ was performed as described.^39^ Mice were elevated by securing the tail 30 cm above the ground with adhesive plaster and isolated from one another using black cardboard. Mice behavior was recorded during 6 min, and time spent immobile during that period was determined by observers blind to mouse treatment.

Tests of object location and novel object recognition, which assess learning and memory,^40^ were conducted as described in a white and square pool^41^ (50 × 50 × 30 cm). A camera was placed over the box for assessment. During the training phase, each mouse was placed in the box and allowed to freely explore two similar objects for 10 min.

In the object location test, one of the objects was moved to a new location at 6 min before the end of test session. The mouse was placed in the box for 10 min. A “location preference index” was calculated by dividing the time spent exploring the object in a novel location by the sum of the times spent exploring the object in novel and familiar locations.

In the novel object recognition test, at 30 min after the end of the training session, one of the objects was replaced with a novel object similar in height and volume, but different in shape and appearance. The mouse was placed in the box for 10 min. A “novel object recognition index” was calculated by dividing the time spent exploring the novel object by the sum of the times spent exploring the novel and familiar objects.

### 2.8 Analysis of gene expression in hippocampi and primary microglia

Brain tissue was prepared from treated mice using PBS perfusion as described,^34^ and hippocampi were isolated. Total RNA was extracted from hippocampi or primary cultures using Trizol (Invitrogen Life Technologies, Carlsbad, CA, USA) according to the manufacturer's instructions. The RNA was converted into complementary DNA using the First Strand cDNA Synthesis Kit (Takara, Shiga, Japan) according to the manufacturer's instructions, then amplified using PCR on a CFX 96 system (Bio‐Rad Laboratories) and specific primers (Table S1). Each sample was test™ed in triplicate. The threshold cycle (Ct) number was determined from the linear phase of the amplification plot using the −ΔΔCt method, and values were normalized against the housekeeping gene β‐actin.

### 2.9 Assays of cytokines, NLRP3 and other key molecules in hippocampi and culture medium

Brain tissue was prepared from treated mice using PBS perfusion as described,^34^ and hippocampi were isolated, flash‐frozen in liquid nitrogen, homogenized, and centrifuged at 1000*g* for 30 min. Medium from primary cultures of microglia was harvested and centrifuged at 1000*g* for 30 min. The supernatant from hippocampi or culture medium was assayed for total protein concentration using the BCA kit (Boster, Wuhan, China), diluted to a final concentration of 1 g/mL, and assayed for IL‐1β, TNF‐α and NLRP3 using enzyme‐linked immunosorbent assays (Boster) according to the manufacturer's protocols. The manufacturer‐specified detection limit was 2 pg/mL for all assays. Supernatants were also assayed for nitric oxide, malondialdehyde, reactive oxygen species and lactate dehydrogenase using commercial kits (BioVision Inc. Milpitas, California, USA) according to the manufacturer's instructions.

### 2.10 Western blotting

Levels of NLRP3, caspase‐1, gasdermin D, IL‐1β, STAT1, phospho‐STAT1 and GAPDH were analyzed as described^11^, ^42^ in hippocampal tissue from treated mice and primary microglia. Fractionated hippocampal lysates were blotted with primary antibodies overnight at 4°C, followed by an appropriate secondary antibody for 30 min at room temperature (Table S2). Finally, membranes were scanned with a V370 scanner (Epson, Shenzhen, China), and bands were quantitated using AlphaEaseFC 4.0 software (Alpha Innotech, Shanghai, China). Original Western Blot images showed in the supplemental files.

### 2.11 Immunohistochemistry against key proteins in mouse hippocampus or primary cultures

Brain tissue was prepared from treated mice using PBS perfusion as described.^34^ Primary microglia and differentiated NSPCs were plated (1 × 10^5^ cells/cm^2^) and fixed with 4% paraformaldehyde (pH 7.2) for 30 min. Hippocampal tissues or cultured cells were permeabilized with 0.5% Triton X‐100 in PBS for 15 min, blocked in 10% donkey serum for 2 h, incubated overnight at 4°C with primary antibodies (Table S3), then 2 h at room temperature with DyLight 549‐conjugated donkey anti‐goat or DyLight 488‐conjugated donkey anti‐mouse secondary antibodies (both 1:300, Jackson ImmunoResearch, West Grove, PA, USA). Samples were counterstained with DAPI (1:10000, Roche, Basel, Swiss) for 5 min and imaged using a fluorescence microscope.

Numbers of microglia were determined using Image J software by counting all Iba1‐expressing cells in 5–6 micrographs at a magnification of 40×. Microglial branching was quantified using Image‐Pro Plus 6.0 (Media Cybernetics, Rockville, MD, USA).

### 2.12 Statistical analysis

All statistical analyses were performed within GraphPad Prism 6 (GraphPad Software, San Diego, CA, USA). Data were reported as mean ± SEM. Shapiro–Wilk test was used to assess data normally distribution. For normally distributed data, pairwise differences were assessed for significance using a paired Student's *t* test for independent samples, one‐way ANOVA was used to analyze differences between multiple groups. Data that did not exhibit a normal distribution were analyzed using Kruskal–Wallis tests between multiple groups. Differences were considered statistically significant if *p* < 0.05.

## 3 RESULTS

### 3.1 IFN‐γ upregulates pro‐inflammatory cytokines and components of the NLRP3 inflammasome

**FIGURE 1:**
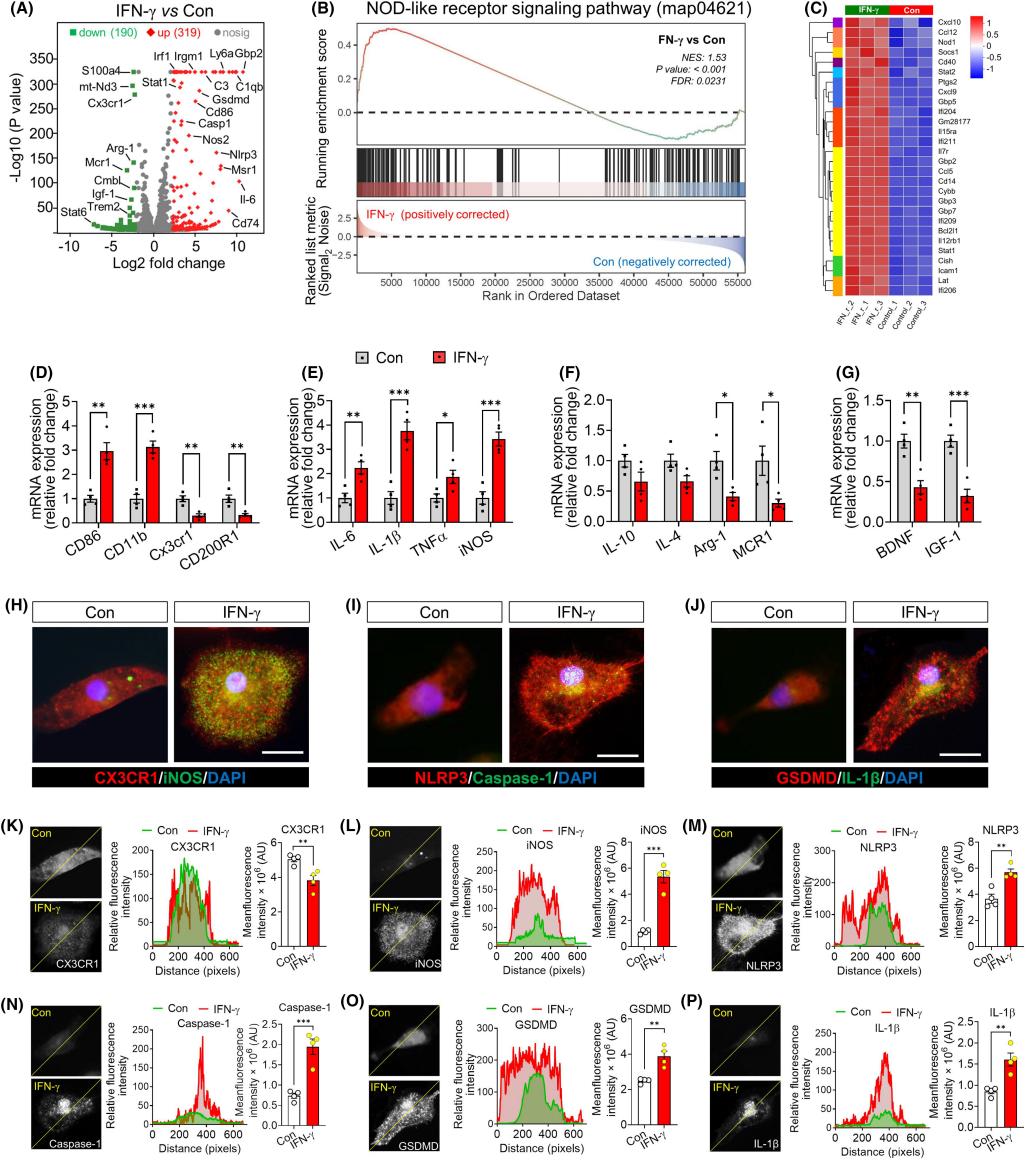
IFN‐γ promotes NLRP3 inflammasome activation and inflammation in primary microglia. (A) Volcano plot of differentially expressed genes (DEGs). Significantly upregulated genes are shown as red dots; significantly downregulated genes, as green dots. (B) Gene set enrichment analysis, indicating enrichment of processes related to NOD‐like receptor signaling pathway (map04621) in the IFN‐γ‐treated microglia compared to that of PBS‐treated microglia. (C) Hierarchical cluster analysis of enriched differentially expressed genes related to NOD‐like receptor signaling pathway. (D–G) Levels of mRNAs encoding microglial markers (D), pro‐inflammatory makers (E), anti‐inflammatory makers (F), and neurotrophic factors (G) were evaluated using q‐PCR. The fold‐expression of each gene was normalized to the control group. (H) Representative immunofluorescence images of CX3CR1 (red) and iNOS (green) expression in IFN‐γ‐primed primary microglia. The nucleus is labeled with DAPI (blue). Scale bar, 10 μm. iNOS, inducible nitric oxide synthase. DAPI, 4′, 6‐diamidino‐2‐phenylindole. (I) Representative immunofluorescence images of NLRP3 (red) and Caspase 1 (green) expression in IFN‐γ‐primed primary microglia. The nucleus is labeled with DAPI (blue). Scale bar, 10 μm. NLRP3, NOD‐like receptor family, pyrin domain containing 3. (J) Representative immunofluorescence images of GSDMD (red) and IL‐1β (green) expression in IFN‐γ‐primed primary microglia. The nucleus is labeled with DAPI (blue). Scale bar, 10 μm. GSDMD, Gasdermin D; IL‐1β, Interleukin‐1 beta. (K–P) Quantification of the relative mean fluorescence density of CX3CR1 (K) and iNOS (L) NLRP3 (M), caspase 1 (N), GSDMD (O) and IL‐1β (P). The corresponding peaks at the bottom of the micrograph represent the dotted‐lines indicating the changes in fluorescence density of each protein. Four, 40× micrographs were taken from each well, and all cells in the field were measured. Data are presented as mean ± SEM (*n* = 4). *p < 0.05, **p < 0.01, ***p < 0.001 by unpaired *t* test. The results of statistical analyses are listed in Table S4.

We prepared primary cultures of microglia from the brains of healthy mice, exposed them to IFN‐γ or PBS for 24 h, then harvested RNA from the two samples and compared the expression of numerous genes using RNA sequencing (Figure S1A). IFN‐γ upregulated 319 genes and downregulated 190 genes in microglia (Figure 1A and Figure S1B). Differentially expressed genes were enriched in pathways related to responses to IFN‐γ, regulation of oxide biosynthesis, regulation of cytokine production, regulation of STAT cascade, secretion of IL‐1β or IL‐18, and activation of microglia (Figure S1C). Most differentially expressed genes were involved in NOD‐like receptor signaling and interactions between cytokines and their receptors (Figure 1B,C).

Using quantitative PCR, we confirmed that IFN‐γ upregulated the markers of microglial activation CD86 and CD11b; the pro‐inflammatory cytokines IL‐1β, IL‐6 and TNF‐α; and inducible nitric oxide synthase (iNOS). At the same time, it downregulated the surface receptors CX3CR1 and CD200R1, the anti‐inflammatory cytokines MCR1 and Arg‐1, and the neurotrophic factors IGF‐1 and brain‐derived neurotrophic factor (Figure 1D–G). By immunostaining primary cultures of microglia, we confirmed that IFN‐γ upregulated iNOS, NLRP3, caspase‐1, gasdermin D and IL‐1β, while downregulating CX3CR1 (Figure 1H–P).

### 3.2 IFN‐γ primes microglia to hyperreact to inflammatory stimulation

**FIGURE 2:**
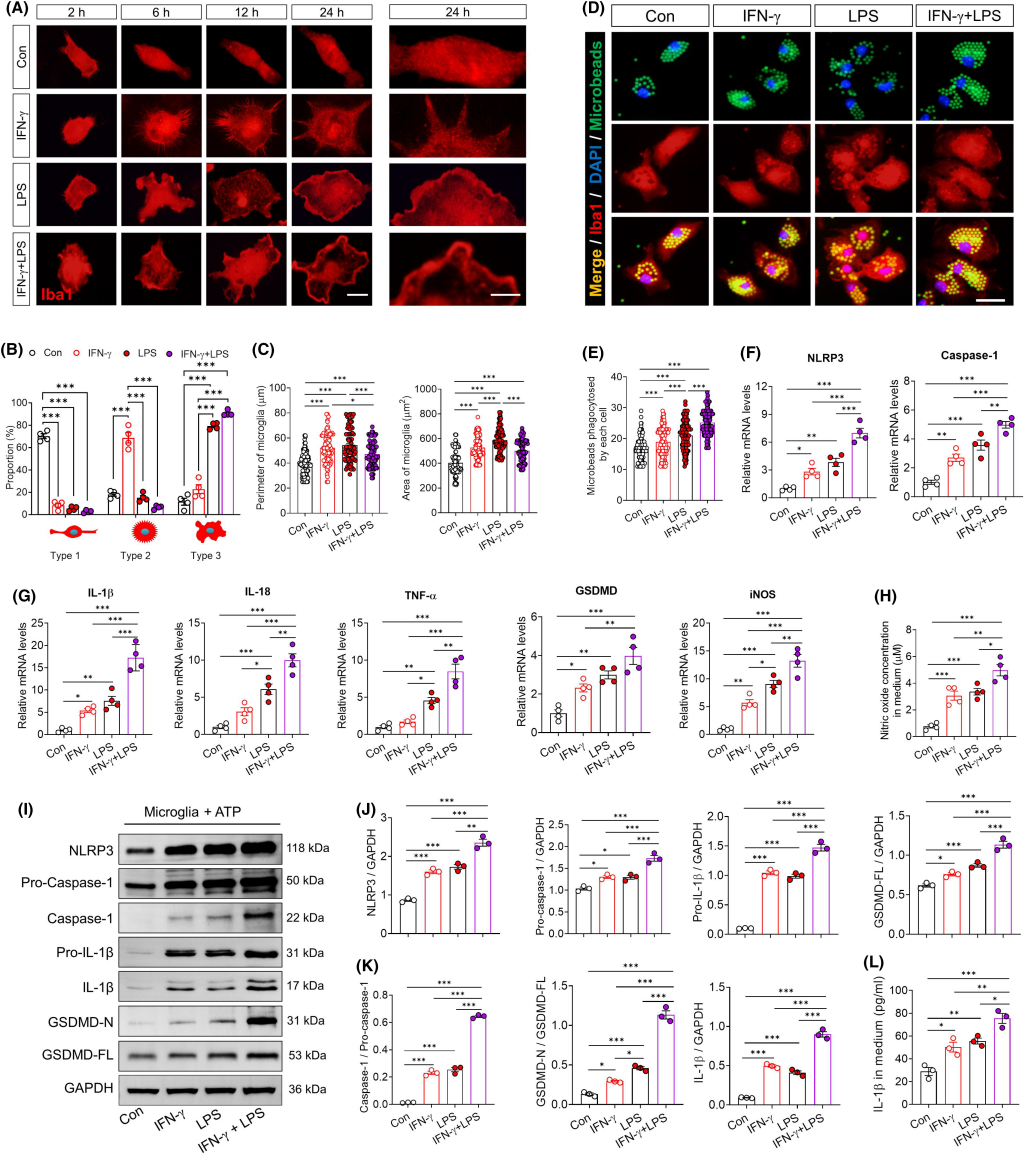
IFN‐γ exaggerates the microglia's response, NLRP3 transcriptional activation and IL‐1β release of microglia to following LPS stimulation. (A) Microglial morphology in response to treatment with PBS, IFN‐γ, LPS or IFN‐γ plus LPS for the indicated times. Microglia were stained with Iba1 by immunocytochemistry. Scale bar, 5 μm. Iba1, Ionized calcium binding adapter molecule 1. (B) Quantification of the proportions of microglia that showed rod‐like (type 1), spiny (type 2), lamellipodium/ameboid (type 3). The bottom row shows representative illustrations of the different microglial morphologies. (C) Quantification of the diameter and area of each microglia treated by PBS, IFN‐γ, LPS or IFN‐γ plus LPS for 24 h. (D) Changes in phagocytic ability of microglia after PBS, IFN‐γ, LPS or IFN‐γ plus LPS treatment. Representative micrographs showing immunocytochemical labeling for Iba1 (red) and engulfed fluorescent microspheres (green) in microglia treated with IFN‐γ or PBS. The nucleus is labeled with DAPI (blue). Scale bar, 10 μm. (E) The histogram represents quantification of the number of engulfed fluorescent microspheres in microglia. Four, 40× micrographs were taken from each well, and all cells in the field were measured. (F, G) Levels of mRNAs encoding NLRP3, Caspase 1, GSDMD, IL‐1β, IL‐18, TNF‐α and iNOS in microglia treated with PBS, IFN‐γ, LPS or IFN‐γ plus LPS for 24 h. Each sample in triplicate. (H) Quantification of the concentration of nitric oxide in medium of primary microglia. (I–K) Western blots showing the changes in the levels of NLRP3, pro‐caspase‐1, caspase‐1, GSDMD‐N, GSDMD, pro‐IL‐1β and IL‐1β of microglia treated with PBS, IFN‐γ, LPS or IFN‐γ plus LPS for 24 h. Each sample was analyzed in triplicate. (L) Quantification of the concentration of IL‐1β in medium of primary microglia. Data are presented as mean ± SEM (*n* = 3–4). *p < 0.05, **p < 0.01, ***p < 0.001 by one‐way ANOVA with Tukey's multiple comparison post hoc test. The results of statistical analyses are listed in Table S5.

Treating primary cultures of microglia with IFN‐γ induced a typical, hedgehog‐like priming morphology of rounded somata with multiple filopodia,^22^, ^27^, ^28^, ^29^ in strong contrast to the spindle shape, small somata and two branches of PBS‐treated microglia. LPS, in contrast, induced ameboid morphology and formation of lamellipodia. The combination of IFN‐γ and LPS led to a condensed and pyroptotic morphology (Figure 2A,B). Treatment with IFN‐γ and LPS alone or together increased the perimeter and area of microglial somata (Figure 2C), as well as the number of microbeads that they phagocytosed (Figure 2D,E). The combination of IFN‐γ and LPS led to smaller perimeter and area of microglial somata, but more phagocytosed microbeads, than either treatment on its own.

IFN‐γ or LPS on their own upregulated NLRP3, caspase‐1, gasdermin D, IL‐1β, IL‐18, TNF‐α and iNOS, and the combination of both treatments upregulated them as well as nitric oxide even more, whether at the level of mRNA (Figure 2F,G) or protein (Figure 2H–L). The greater effects of dual stimulation than with LPS alone suggest that IFN‐γ primes microglia by promoting activation of the NLRP3 inflammasome and secretion of IL‐1β.

**FIGURE 3:**
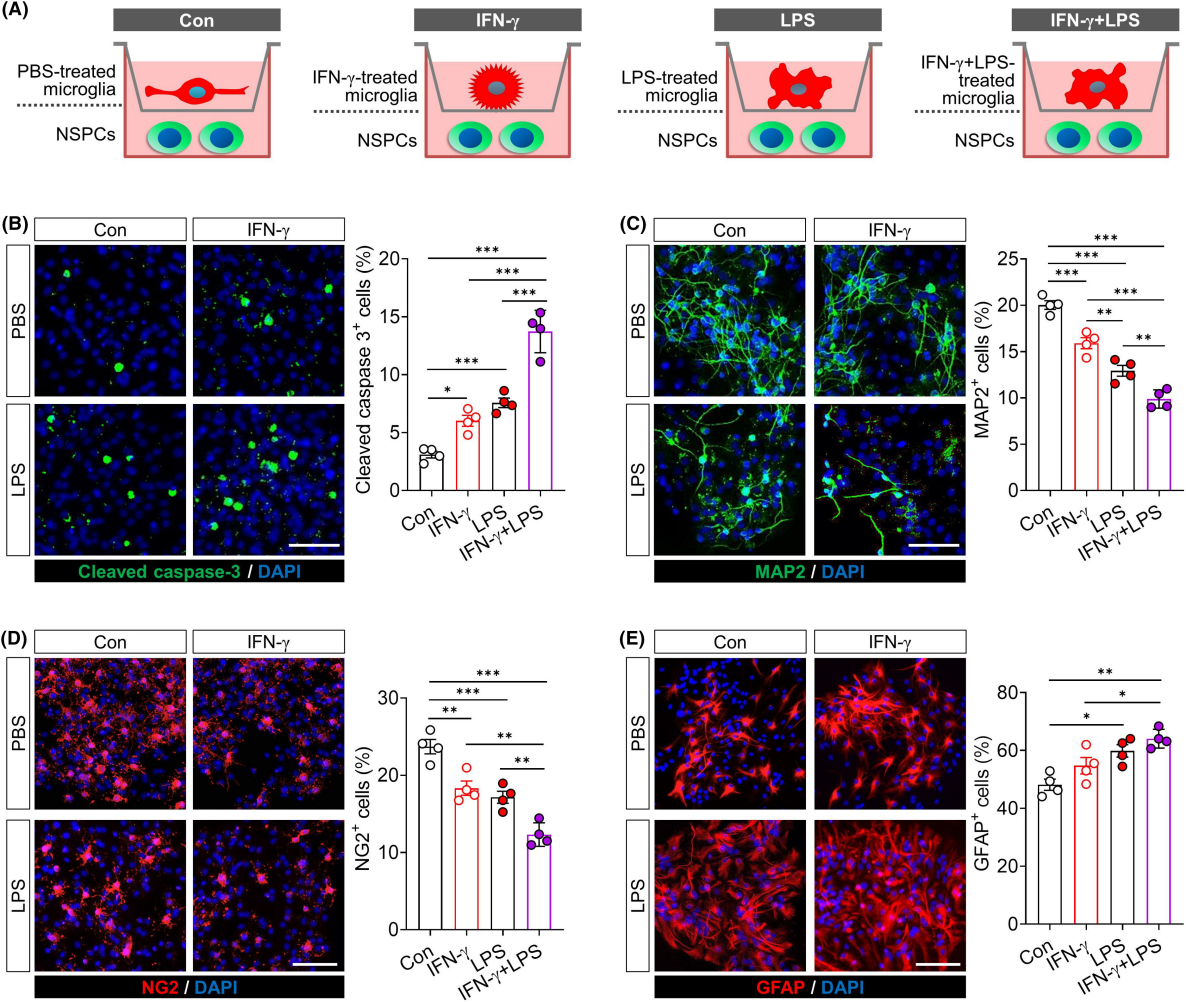
IFN‐γ exaggerates the immunotoxicity of microglia on neural stem cells. (A) Experimental scheme to research the effects of microglia exposed to PBS, IFN‐γ, LPS or IFN‐γ plus LPS for 24 h on neural stem/precursor cells (NSPCs). NSPCs were co‐cultured with above microglia by transwell. (B) Effects of microglia exposed to PBS, IFN‐γ, LPS or IFN‐γ plus LPS for 24 h on survival of NSPCs. Apoptotic cells were labeled with cleaved caspase‐3. The nucleus is labeled with DAPI (blue). Scale bar is 20 μm. Statistical graphs showed the percentage of cleaved caspase‐3^+^ cells out of DAPI^+^ cells. (C) Effects of microglia exposed to PBS, IFN‐γ, LPS or IFN‐γ plus LPS for 7 days on neurogenesis. Neurons were labeled with MAP2. The nucleus is labeled with DAPI (blue). Scale bar is 20 μm. Statistical graphs showed the percentage of MAP2^+^ cells out of DAPI^+^ cells. (D) Effects of microglia exposed to PBS, IFN‐γ, LPS or IFN‐γ plus LPS for 7 days on oligodendrogenesis. Oligodendrocyte precursor cells were labeled with NG2. The nucleus is labeled with DAPI (blue). Scale bar is 20 μm. Statistical graphs showed the percentage of NG2^+^ cells out of DAPI^+^ cells. (E) Effects of microglia exposed to PBS, IFN‐γ, LPS or IFN‐γ plus LPS for 7 days on NSPCs differentiate into astrocytes. Astrocyte were labeled with GFAP. The nucleus is labeled with DAPI (blue). Scale bar is 20 μm. Statistical graphs showed the percentage of GFAP^+^ cells out of DAPI^+^ cells. Data are presented as mean ± SEM (*n* = 4). *p < 0.05, **p < 0.01, ***p < 0.001 by one‐way ANOVA with Tukey's multiple comparison post hoc test. The results of statistical analyses are listed in Table S6.

Consistent with the priming ability of IFN‐γ, we found that primary microglia that had been stimulated with both LPS and IFN‐γ were more effective at inducing apoptosis in co‐cultured NSPCs than microglia stimulated with either treatment, based on the increases in the abundance of cells expressing cleaved caspase‐3. At the same time, the dual‐treated microglia were also more effective at shifting the differentiation of co‐cultured NSPCs away from neurons or oligodendrocyte precursors and toward astrocytes, based on increases in the abundance of cells expressing the astrocyte marker GFAP and concomitant decreases in numbers of cells expressing the neuronal marker MAP2 or oligodendrocyte marker NG2 (Figure 3).

### 3.3 IFN‐γ acts via STAT1 to activate the NLRP3 inflammasome in microglia in vitro

**FIGURE 4:**
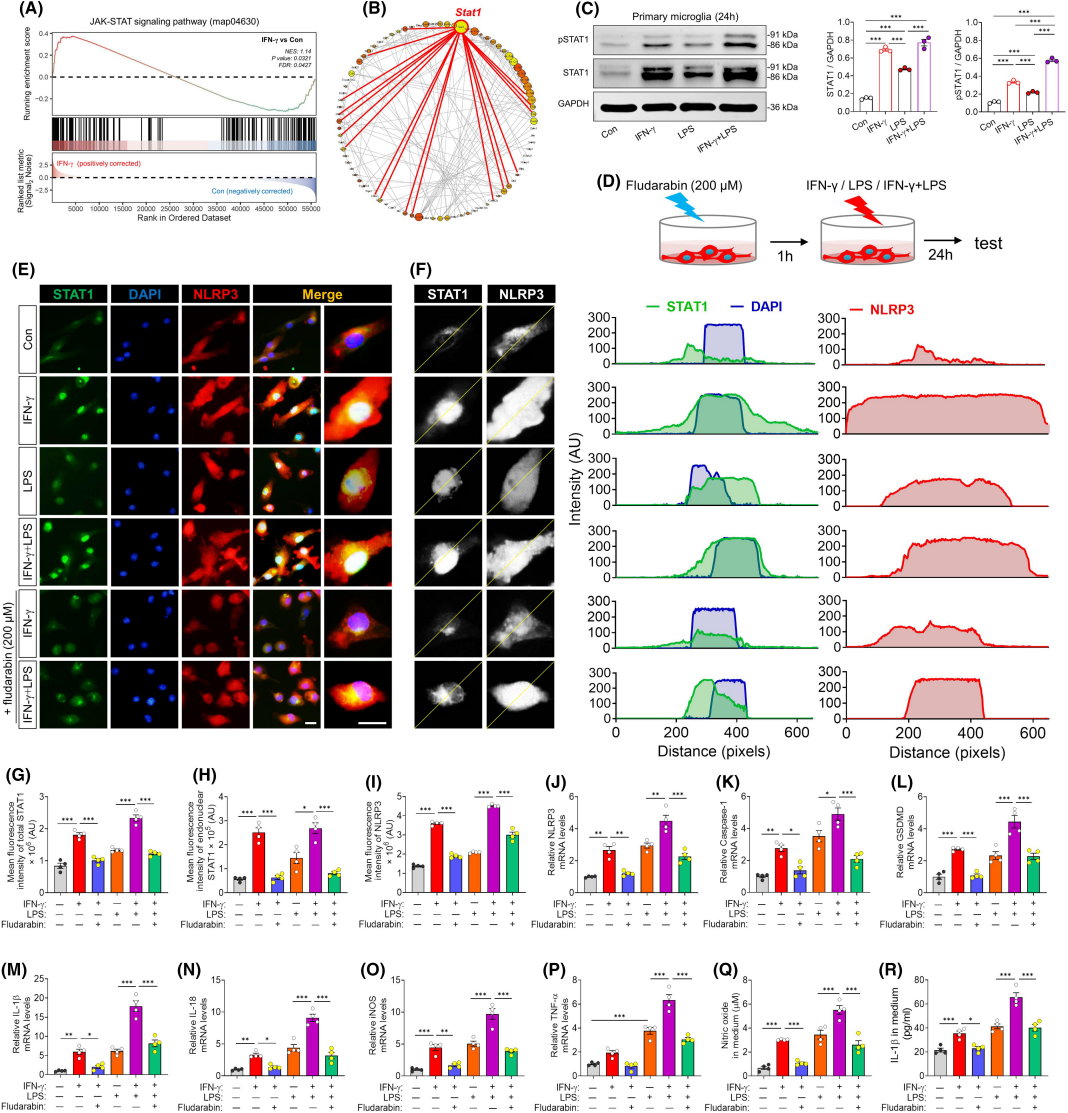
Blockade of STAT1 signaling partially abolished the NLRP3 inflammasome activation in IFN‐γ‐treated microglia. (A) Gene set enrichment analysis, indicating enrichment of processes related to Jak–STAT signaling pathway (map04630) in the IFN‐γ‐treated microglia compared to that of PBS‐treated microglia. (B) Interaction network analysis of proteins encoded by differentially expressed genes (DEGs) between IFN‐γ‐primed microglia and PBS‐treated microglia. (C) Western blots showing the changes in the levels of STAT1 and pSTAT1 in microglia treated with PBS, IFN‐γ, LPS or IFN‐γ plus LPS for 24 h. Each sample was analyzed in triplicate. (D) Experimental scheme to research the effects of STAT1 inhibitor fludarabin on microglia exposed to IFN‐γ or IFN‐γ plus LPS for 24 h. (E) Representative immunofluorescence images of NLRP3 (red) and STAT1 (green) expression in microglia exposed to PBS, IFN‐γ, LPS or IFN‐γ plus LPS for 24 h in present or absent of STAT1 inhibitor fludarabin. The nucleus is labeled with DAPI (blue). Scale bar, 10 μm. (F–I) Quantification of the relative mean fluorescence density of NLRP3 and nuclear translocation of STAT1. The corresponding peaks at the right of the micrograph represent the dotted‐lines indicating the changes in fluorescence density of each protein. Four, 40× micrographs were taken from each well, and all cells in the field were measured. (J–P) Levels of mRNAs encoding NLRP3, Caspase 1, GSDMD, IL‐1β, IL‐18, TNF‐α or iNOS in microglia treated with PBS, IFN‐γ, LPS or IFN‐γ plus LPS for 24 h in present or absent of STAT1 inhibitor fludarabin. Each sample in triplicate. (Q–R) Quantification of the concentration of nitric oxide and IL‐1β in medium of primary microglia. Data (C–L) are presented as mean ± SEM (*n* = 4). *p < 0.05, **p < 0.01, ***p < 0.001 by one‐way ANOVA with Tukey's multiple comparison post hoc test. The results of statistical analyses are listed in Table S7.

In silico analyses comparing genes differentially expressed between primary microglia treated with IFN‐γ or PBS identified the transcription STAT1 as a potential driver of the interferon's effects (Figure 4A,B and Figure S2A–D). STAT1 is phosphorylated by receptor kinases such as JAK1 and JAK2, inducing its dimerization and translocation into the nucleus.^43^ We confirmed that treating primary microglia with IFN‐γ led to higher levels of total STAT1 and phospho‐STAT1 (Figure 4C). The STAT1 inhibitor fludarabin antagonized the ability of IFN‐γ to upregulate NLRP3, caspase‐1, gasdermin D, IL‐1β, IL‐18, TNF‐α, and iNOS, and thereby inhibited its ability to prime these microglial responses to LPS (Figure 4D–R).

**FIGURE 5:**
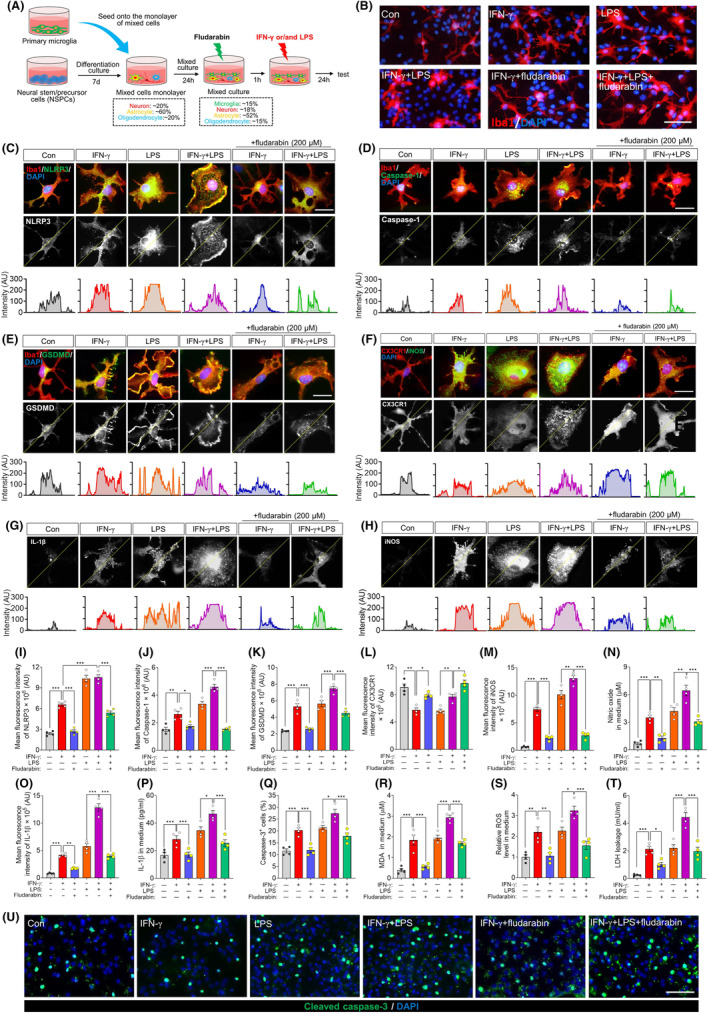
Effects of STAT1 inhibitor on NLRP3 inflammasome activation and inflammatory mediators of IFN‐γ‐treated microglia in a mixed culture system. (A) Experimental scheme detailing co‐culture of primary microglia with neuron, astrocyte and oligodendrocyte. The neural stem/progenitor cells (NSPCs) are induced to differentiate into neuron, astrocyte and oligodendrocyte. These cells were attached on the bottom of plate to form a monolayer. After that, primary microglia are cultured on the monolayer of mixed cells for 24 h. They then received PBS, IFN‐γ, LPS or IFN‐γ plus LPS stimulation for another 24 h in present or absent of STAT1 inhibitor fludarabin pretreatment. (B) Representative immunofluorescence images showing morphological differences of microglia cultured on monolayer of mixed cells among each group. Microglia were labeled with Iba1 (red), and nucleus labeled with DAPI (blue). Scale bar, 20 μm. (C–E) Representative immunofluorescence images of NLRP3 (C), Caspase1 (D), and GSDMD (E) (green) and Iba1 (red) expression in microglia exposed to PBS, IFN‐γ, LPS or IFN‐γ plus LPS for 24 h in present or absent of STAT1 inhibitor fludarabin. The nucleus is labeled with DAPI (blue). Scale bar, 10 μm. The corresponding peaks at the bottom of the micrograph represent the dotted‐lines indicating the changes in fluorescence density of NLRP3 (C), Caspase1 (D), and GSDMD (E). (F, H) Representative immunofluorescence images of CX3CR1 (red) and iNOS (green) expression in microglia exposed to PBS, IFN‐γ, LPS or IFN‐γ plus LPS for 24 h in present or absent of STAT1 inhibitor fludarabin. The nucleus is labeled with DAPI (blue). Scale bar, 10 μm. The corresponding peaks at the bottom of the micrograph represent the dotted‐lines indicating the changes in fluorescence density of CX3CR1 (F) and iNOS (H). (G) Representative immunofluorescence images of IL‐1β in microglia exposed to PBS, IFN‐γ, LPS or IFN‐γ plus LPS for 24 h in present or absent of STAT1 inhibitor fludarabin. The corresponding peaks at the bottom of the micrograph represent the dotted‐lines indicating the changes in fluorescence density of IL‐1β. (I–K) Quantification of the relative mean fluorescence density of NLRP3 (I), Caspase1 (J) and GSDMD (K) in each group. Four, 40× micrographs were taken from each well, and all cells in the field were measured. (L, M) Quantification of the relative mean fluorescence density of CX3CR1 (L) and iNOS (M) in each group. Four, 40× micrographs were taken from each well, and all cells in the field were measured. (N) Quantification of the concentration of nitric oxide in medium of each group. (O) Quantification of the relative mean fluorescence density of IL‐1β in each group. (P) Quantification of the concentration of IL‐1β in medium of each group. (Q) Quantification of percentage of cleaved caspase‐3^+^ cells out of DAPI^+^ cells. (R–T) Quantification of the levels of MDA, ROS and LDH in medium of each group. (U) Effects of microglia exposed to PBS, IFN‐γ, LPS or IFN‐γ plus LPS for 24 h in present or absent of STAT1 inhibitor fludarabin on survival of NSPCs. Apoptotic cells were labeled with cleaved caspase‐3. The nucleus is labeled with DAPI (blue). Scale bar is 50 μm. Data are presented as mean ± SEM (*n* = 4). *p < 0.05, **p < 0.01, ***p < 0.001 by one‐way ANOVA with Tukey's multiple comparison post hoc test. The results of statistical analyses are listed in Table S8.

We wished to verify the effects of IFN‐γ and the dependence on STAT1 in a culture system that better reflects the complex cellular environment of the brain. Therefore we explored the effects of stimulating primary microglia with IFN‐γ or LPS in mixed cultures containing microglia, neurons, astrocytes, and oligodendrocytes (Figure 5A). In these mixed cultures, microglia grew numerous thin branches (Figure 5B), in contrast their behavior in monoculture.^44^ As in the simpler co‐culture system above, treating these mixed cultures with INF‐γ activated microglia and primed them to strengthen their responses to LPS, while fludarabin antagonized these effects (Figure 5C–E,I–K). Indeed, fludarabin partially reversed the effects of INF‐γ or LPS on expression of CX3CR1, iNOS and IL‐1β (Figure 5F–H,L–P). It also antagonized the effects of INF‐γ or LPS on levels of apoptosis, based on numbers of cells expressing cleaved caspase‐3 and on levels of malondialdehyde, reactive oxygen species and lactate dehydrogenase in culture medium (Figure 5Q–U).

### 3.4 IFN‐γ acts via STAT1 to activate the NLRP3 inflammasome in hippocampal microglia in vivo

**FIGURE 6:**
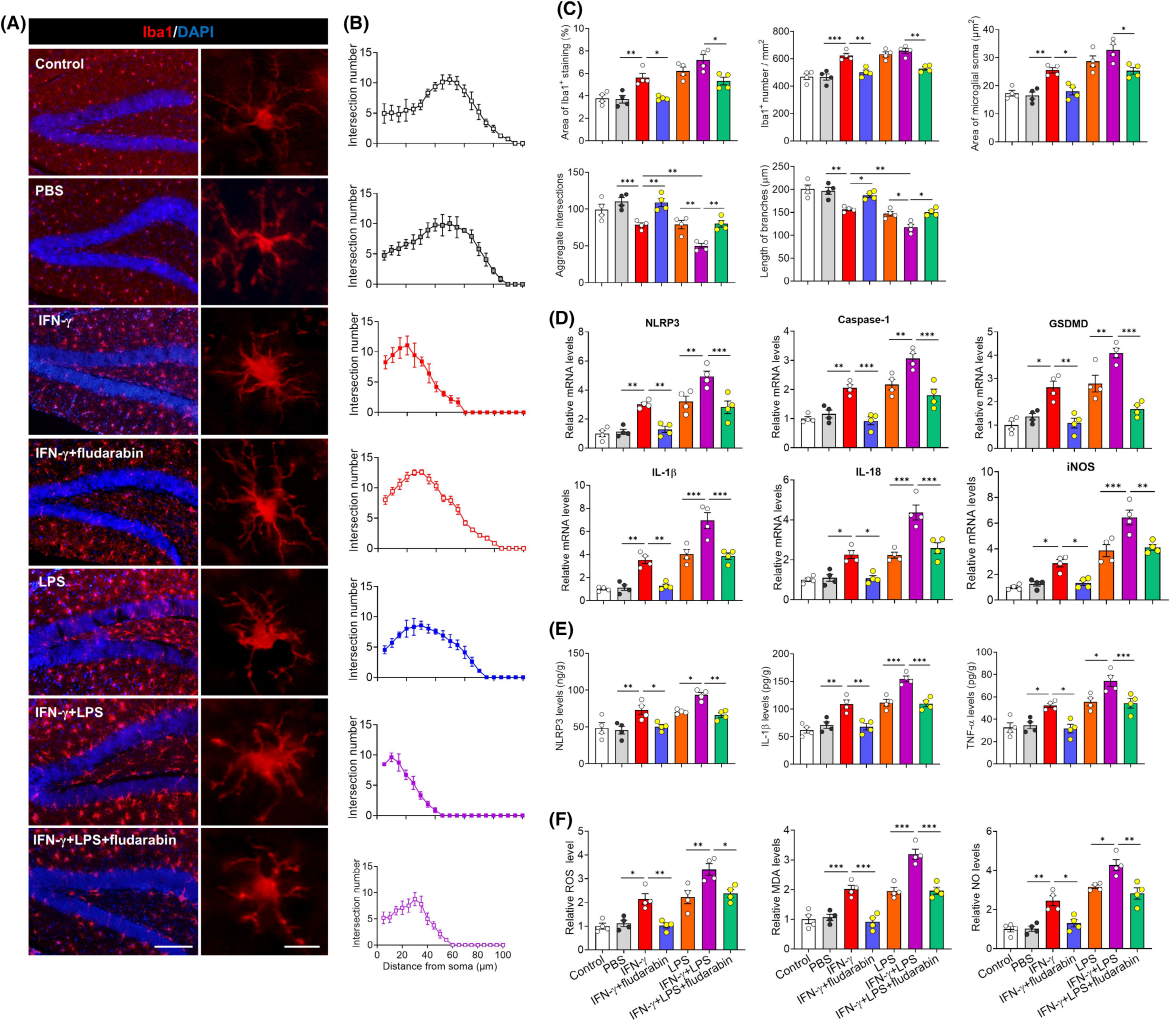
Blockade of STAT1 signaling partially abolished the priming of microglia and NLRP3 inflammasome activation in hippocampus of IFN‐γ‐injected mice. (A) Representative sections of hippocampus from mice that were injected with PBS, IFN‐γ, LPS or IFN‐γ plus LPS injection in present or absent of STAT1 inhibitor fludarabin were immunostained against “ionized calcium binding adapter molecule 1” (Iba1) as a marker of microglia. The nucleus is labeled with DAPI (blue). Scale bar, 100 μm. The enlarged graphs on the middle are the microglia in hippocampal dentate gyrus. Scale bar, 100 μm. The enlarged graphs on the right are the typical morphology of microglia in hippocampus. Scale bar, 15 μm. (B) The number of intersections of microglial branches were evaluated by Sholl analysis in the hippocampus of each group mice. (C) Quantification of the area of Iba1^+^ staining, number of microglia, area of microglial soma, accumulated intersections of microglial branches and length of microglial branches in hippocampus. Results come from five slices of hippocampus (at 40 × magnification) from each of four mice per condition. Each dot represents the average of all micrographs for one mouse. (D) Levels of mRNAs encoding NLRP3, Caspase 1, GSDMD, IL‐1β, IL‐18 or iNOS in hippocampus of mice injected with PBS, IFN‐γ, LPS or IFN‐γ plus LPS in present or absent of STAT1 inhibitor fludarabin. Each sample in triplicate. (E) Quantification of the concentration of NLRP3, IL‐1β and TNF‐α in hippocampus of mice injected with PBS, IFN‐γ, LPS or IFN‐γ plus LPS in present or absent of STAT1 inhibitor fludarabin. (F) Quantification of the levels of MDA, ROS and NO in hippocampus of mice injected with PBS, IFN‐γ, LPS or IFN‐γ plus LPS in present or absent of STAT1 inhibitor fludarabin. Data are presented as mean ± SEM (*n* = 4). *p < 0.05, **p < 0.01, ***p < 0.001 by one‐way ANOVA with Tukey's multiple comparison post hoc test. The results of statistical analyses are listed in Table S9.

Finally, we wished to verify these results from mixed cultures in the functioning brain. Mice were injected directly in the brain with IFN‐γ alone, LPS alone, or both. In all three cases, microglia in the hippocampus adopted an activated morphology (Figure 6A,B), while their density and surface area within the tissue increased (Figure 6C). The length and intersections of microglial branches decreased, and this effect was stronger in animals treated with both IFN‐γ and LPS (Figure 6C). Intraperitoneal injection of fludarabin antagonized the effects of IFN‐γ and LPS.

**FIGURE 7:**
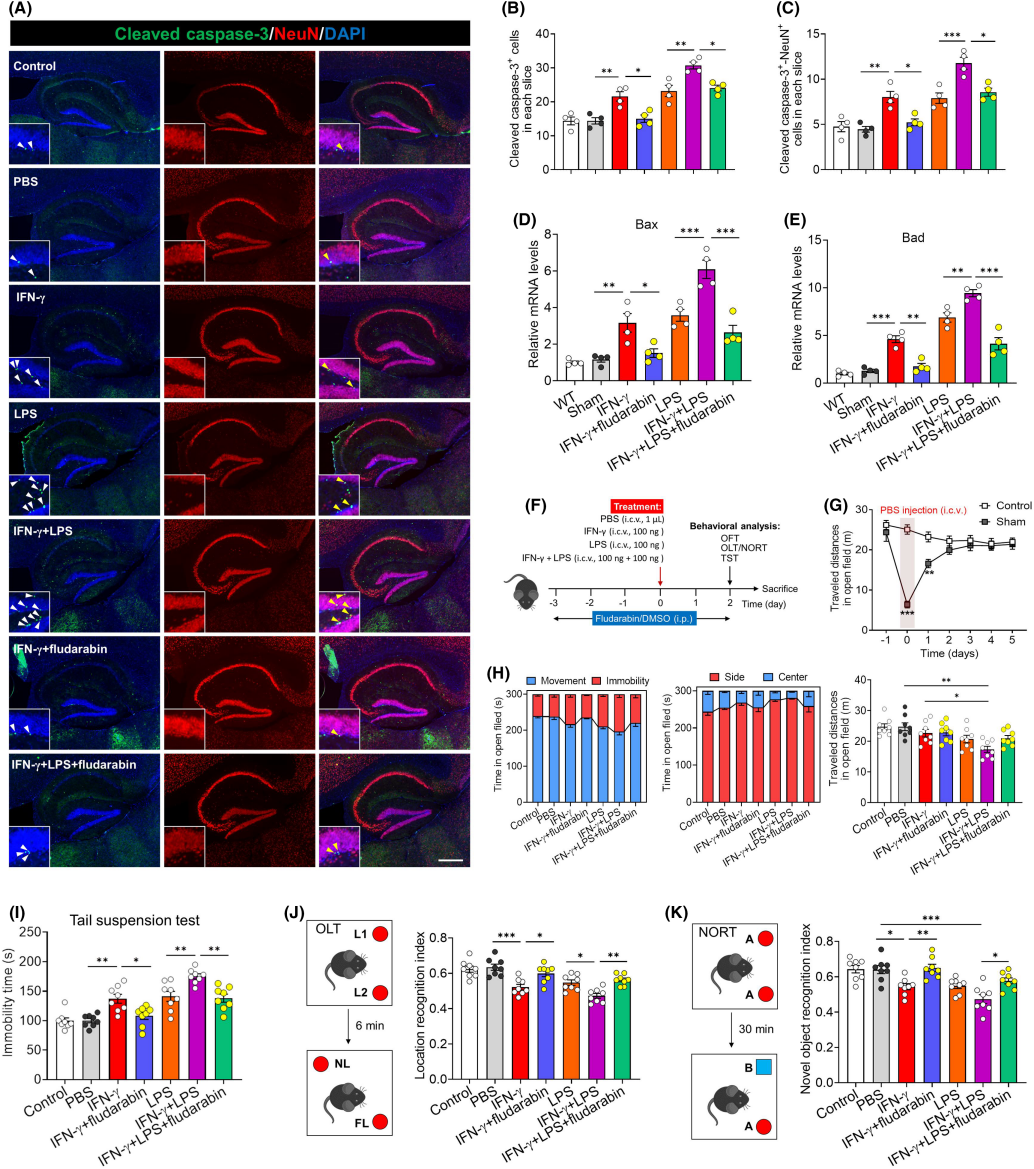
Blockade of STAT1 signaling partially abolished the IFN‐γ‐induced impairment in hippocampal neuron and behavior. (A) Representative sections of hippocampus from mice that were injected with PBS, IFN‐γ, LPS or IFN‐γ plus LPS injection in present or absent of STAT1 inhibitor fludarabin were immunostained against NeuN (red) as a marker of neuron. Apoptotic cells were labeled with cleaved caspase‐3 (green). The nucleus is labeled with DAPI (blue). Scale bar, 100 μm. Scale bar, 100 μm. The enlarged graphs on the left bottom are the cleaved caspase‐3^+^‐NeuN^+^ cells (as shown by white arrows). (B, C) Quantification of the number of cleaved caspase‐3^+^ cells and the cleaved caspase‐3^+^‐NeuN^+^ cells in hippocampus of mice injected with PBS, IFN‐γ, LPS or IFN‐γ plus LPS injection in present or absent of STAT1 inhibitor fludarabin. Results come from five slices of hippocampus (at 40× magnification) from each of four mice per condition. Each dot represents the average of all micrographs for one mouse. (D, E) Levels of mRNAs encoding Bax and Bad in hippocampus of mice injected with PBS, IFN‐γ, LPS or IFN‐γ plus LPS in present or absent of STAT1 inhibitor fludarabin. Each sample in triplicate. (F) Experimental scheme detailing behavioral analysis within 48 h after PBS, IFN‐γ, LPS or IFN‐γ plus LPS injection in present or absent of STAT1 inhibitor fludarabin. IFN‐γ, interferon gamma; LPS, lipopolysaccharide; i.c.v., intracerebroventricular injection; i.p., intraperitoneal injection; DMSO, dimethylsulfoxide; OFT, open field test; OLT, object location test; NORT, novel object recognition test; TST, tail suspension test. (G) Evaluation of locomotion activity levels of C57BL/6J mice injected PBS into the lateral ventricle within 5 days. Sham, intracerebroventricular injection PBS. (H) Quantization of time spent in movement and immobility, time spent in the center and side, and distance traveled by mice in the OFT injected with PBS, IFN‐γ, LPS or IFN‐γ plus LPS injection in present or absent of STAT1 inhibitor fludarabin. (I) Quantization of immobility time of each group mice in tail suspension test. (J) Schematic for object location test and location recognition index was compared between the groups. The location preference index was calculated by the time spent exploring object in novel location (NL) out of the sum of the time spent exploring object in novel location (NL) and familiar location (FL). (K) Schematic for novel object recognition test and novel object recognition index was compared between the groups. The novel object recognition index was calculated by the time spent exploring object in novel object (NO) out of the sum of the time spent exploring novel object (NO) and familiar object (FO). Data are presented as mean ± SEM (*n* = 4 (A–E), *n* = 8 (F–L)). *p < 0.05, **p < 0.01, ***p < 0.001 by one‐way ANOVA with Tukey's multiple comparison post hoc test. The results of statistical analyses are listed in Table S10.

Also consistent with our culture experiments, we found that treating mice with IFN‐γ alone or together with LPS upregulated NLRP3, caspase‐1, gasdermin D, IL‐1β, IL‐18 and iNOS in hippocampus (Figure 6D), while fludarabin antagonized the effects of these treatments on expression of NLRP3, IL‐1β, and TNF‐α as well as on levels of reactive oxygen species, malondialdehyde and nitric oxide (Figure 6E,F). Similarly, fludarabin partially reversed the ability of IFN‐γ or LPS to promote apoptosis in the hippocampus, based on the number of cells expressing cleaved caspase‐3, the number of cells expressing both cleaved caspase‐3 and the neuronal marker NeuN, and the total levels of Bax and Bad in hippocampus tissue of mice (Figure 7A–E).

### 3.5 IFN‐γ acts via STAT1 to induce behavioral deficits in mice

To examine whether the observed effects of IFN‐γ and its dependence on STAT1 might help explain the associations between microglial priming and neurological disorders, we evaluated the behavior of the mice treated as described in Section 3.4 using a battery of tests (Figure 7F). We performed these evaluations at 48 h after injection because by this time, the distance traveled by PBS‐injected mice in the open field test was similar to that of uninjected controls (Figure 7G). IFN‐γ or LPS reduced performance in the open field test, and the combination of IFN‐γ and LPS reduced it even more, while fludarabin treatment antagonized these effects (Figure 7H). Similar results were observed in tail suspension test (Figure 7I), the object location test (Figure 7J) and novel object recognition test (Figure 7K).

These results suggested that blockade of STAT1 signaling partially abolished the IFN‐γ‐ or IFN‐γ plus LPS‐induced anxiety, depression and impairments in learning and memory.

## 4 DISCUSSION

Here we provide what appear to be the first molecular details of how IFN‐γ primes microglia. Our results suggest that it acts through STAT1 to promote activation of the NLRP3 inflammasome and secretion of IL‐1β. These insights may help future efforts to elucidate how microglial priming contributes to disease. For example, such microglial priming caused by α‐synuclein can induce production of pro‐inflammatory factors to amplify oxidative damage and accelerate the aggregation of amyloid β in mouse models of Alzheimer's disease.^45^, ^46^ Microglial priming has also been implicated in neuropsychiatric diseases and prion diseases.^47^, ^48^, ^49^

IFN‐γ in our experiments altered gene expression in ways that are expected to trigger inflammatory and immune responses and that enhance microglial responses to pro‐inflammatory stimuli such as LPS, consistent with the known effects of microglial priming.^50^ IFN‐γ stimulation induces particular microglia transcriptional feature, as has been previously concluded: the specific surrounding microenvironment and immune stimulation induce an adaptive microglial phenotype.^51^ It also induced apoptosis of NSPCs and shifted their differentiation away from neurons and toward astrocytes. In short, IFN‐γ induced microglia in our experiments to behave like “disease‐associated microglia.”^52^ We observed similar effects of IFN‐γ in simple cultures of primary microglia; co‐cultures of microglia and NSPCs; a more physiological mixed culture system containing microglia, neurons, astrocytes and oligodendrocytes; and mice. Therefore, our mechanistic findings are likely to be robust. They are also likely to be clinically relevant, given that our mouse studies linked STAT1‐dependent priming by IFN‐γ to behavioral deficits. Indeed, our findings are consistent with previous studies linking microglial priming by IFN‐γ to deficits in processing of neural information^53^ and hippocampal neurogenesis,^54^ which in turn have been linked to depression, anxiety, and cognitive impairment.^54^

The NLRP3 inflammasome appears to be a major driver of neuroinflammation, neurodegenerative diseases and psychiatric disorders that have been linked to microglial hyperreactivity.^55^ In our experiments in culture and mice, IFN‐γ upregulated several molecules involved in the inflammasome and its activation, including NLRP3, caspase‐1, gasdermin D, IL‐1β, and IL‐18. Such upregulation was even higher when IFN‐γ was co‐administered with LPS, reflecting a priming effect on microglia. Activation of the NLRP3 inflammasome induces the maturation of caspase‐1, leading to the release of pro‐inflammatory cytokines such as IL‐1β and IL‐18, which ultimately trigger pyroptosis.^56^ Our data suggest that the primed activation of the NLRP3 inflammasome also leads to secondary inflammatory cascades involving TNF‐α, nitric oxide and potentially other factors, which would help explain the observed increases in malondialdehyde, reactive oxygen species and iNOS.^57^

We found that IFN‐γ downregulated the receptors CX3CR1 and CD200R1 on the surface of microglia. The downregulation of CX3CR1 and CD200R1, which constitute the homeostatic signature of microglia and act as microglia surface receptor, leads to microglia NLRP3 activation more easily and susceptibility to subsequent inflammatory challenges.^58^ Indeed, these two receptors are downregulated in primed, disease‐associated microglia.^52^ Future research should explore how surface receptors upstream of the inflammasome, which may include Toll‐like receptors,^59^ regulate microglial priming.

Future research should also verify and extend our findings that STAT1 helps mediate microglial priming by IFN‐γ, which is consistent with a previous study showing that after activation by the interferon, STAT1 remains bound to promoters and enhancers of the genes encoding TNF‐α, IL‐β and other pro‐inflammatory factors, inducing chronic inflammatory responses.^60^ Since activation of nuclear factor (NF)‐κB has been implicated in microglial priming,^61^ it would be interesting to explore whether the STAT1 and NF‐κB pathways of transcriptional regulation interact during priming. In support of this idea, IFN‐γ has been shown to induce cross‐talk between STAT1 and NF‐κB that leads to inflammation and neurodegeneration.^62^, ^63^ Such cross‐talk may be regulated by double‐stranded RNA‐activated protein kinase (PKR).^63^

Regrettably, to avoid the interference of female sex steroid hormones and estrous cycle, only male mice were used in this study. Due to the sexual dimorphism and potential sex bias occurs in neuron structure, neural reward function and motivation, the sex difference in our research needs to be explored further.

## 5 CONCLUSIONS

Our experiments in simple and mixed culture systems as well as in mice suggest that IFN‐γ potentiates or primes microglial responses to inflammatory stimuli by activating STAT1 to turn on the expression of genes involved in the NLRP3 inflammasome and its activation. Targeting IFN‐γ and/or STAT1 may be a strategy to treat neurodegenerative diseases and psychiatric disorders that involve microglial hyperactivity.

## AUTHOR CONTRIBUTIONS

Jinqiang Zhang and Zili You conceived and designed the study. Jinqiang Zhang analyzed transcriptomic data. Xiaomei Zhang, Hui He and Gaojie Xu performed cell culture, real‐time PCR, western blotting, and stereotactic brain injection, and they evaluated animal behavior. Haili He, Liangyuan Li, Chengyan Yang and Yue Liu performed immunostaining and enzyme‐linked immunosorbent assays, and they analyzed the resulting data. Haili He and Xiaomei Zhang wrote the manuscript, which all authors read and approved.

## FUNDING INFORMATION

This work was supported by the University Science and Technology Innovation Team of the Guizhou Provincial Department of Education [Qian‐jiao‐ji (2023)071], the Scientific and Technological Innovation Project of the Chinese Academy of Chinese Medical Science (CI2021B013), the Guizhou Provincial Science and Technology Project [ZK(2022)505], the National and Provincial Scientific and Technological Innovation Talent Team of the Guizhou University of Traditional Chinese Medicine [Gui‐zhong‐yi TD he‐zi (2022)003], and the Sichuan Science and Technology Program (2020YJ0225).

## CONFLICT OF INTEREST STATEMENT

The authors declare that they have no competing interests.

## Supporting information


Data S1.

## Data Availability

Data can be obtained from the corresponding authors upon reasonable request.
