# Supplementary material for: Microglial priming by IFN‐γ involves STAT1‐mediated activation of the NLRP3 inflammasome
Source: CNS Neurosci Ther. 2024 Oct 11;30(10):e70061. doi: 10.1111/cns.70061 (PMC11468839; doi:10.1111/cns.70061)
Supplement: Supplementary file 1 — Data S1. [file CNS-30-e70061-s001.zip › supplementary figuers.pdf]

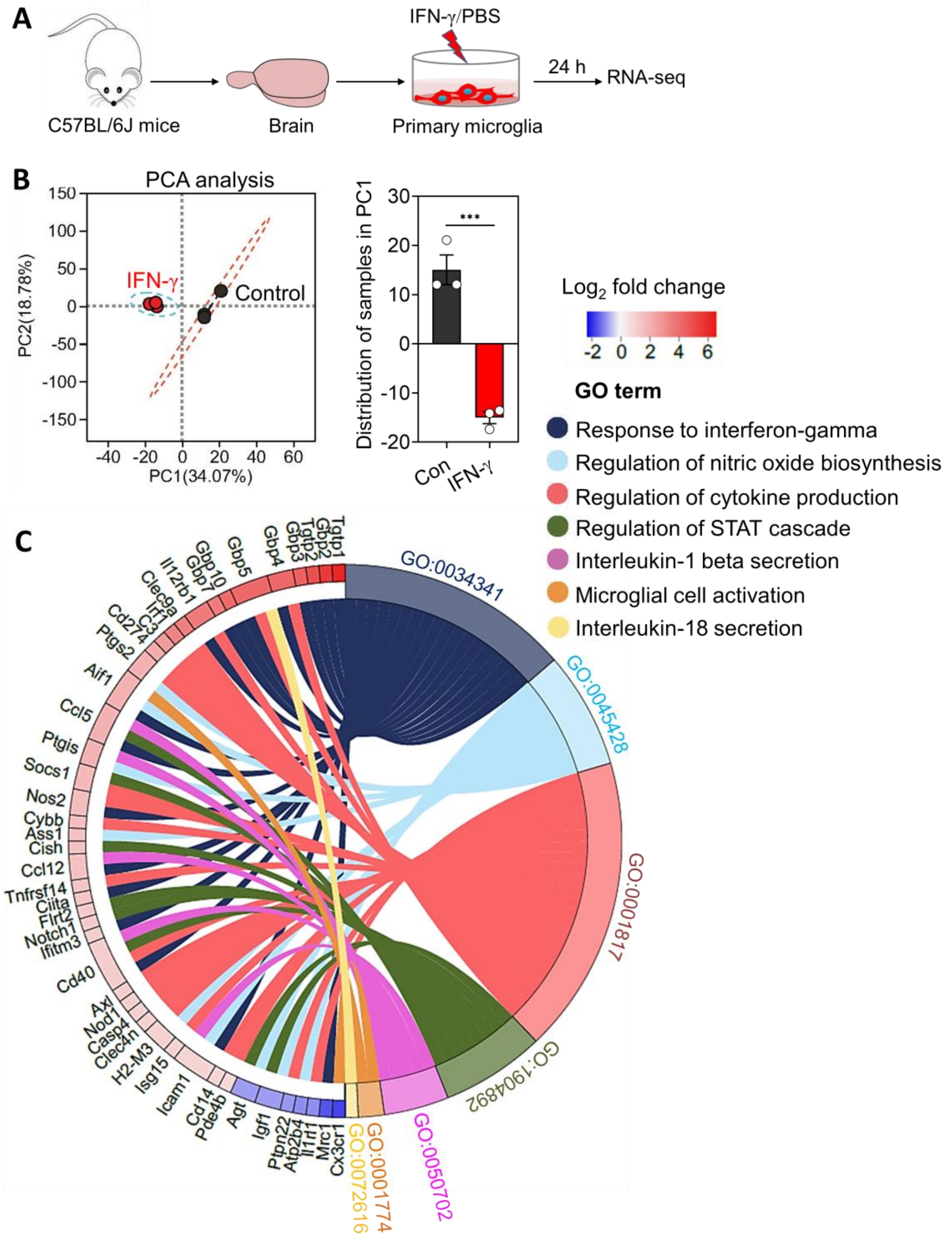

Fig. S1 Transcriptome analysis of IFN-γ-primed primary microglia.

(A) Experimental scheme detailing RNA sequencing (RNS-seq) for phosphate buffer solution (PBS)- or interferon-gamma (IFN-γ)-treated primary microglia.

(B) Principal component analysis to identify clusters of transcriptomes of control or IFN-γ-treated primary microglia. PC, principal component.

(C) Circos plot showing the connectivity map derived from the pairwise comparison of transcriptome datasets. The connectivity between DEGs (left) and Gene Ontology (GO) term (right) are shown in different color. Each line represents a pairwise dataset overlap, which was determined using Gene Set Enrichment Analysis and filtered by $P < 0.05$ and normalized enrichment score > 1.5. The change in the expression of DEGs was quantified as the log2 fold change.

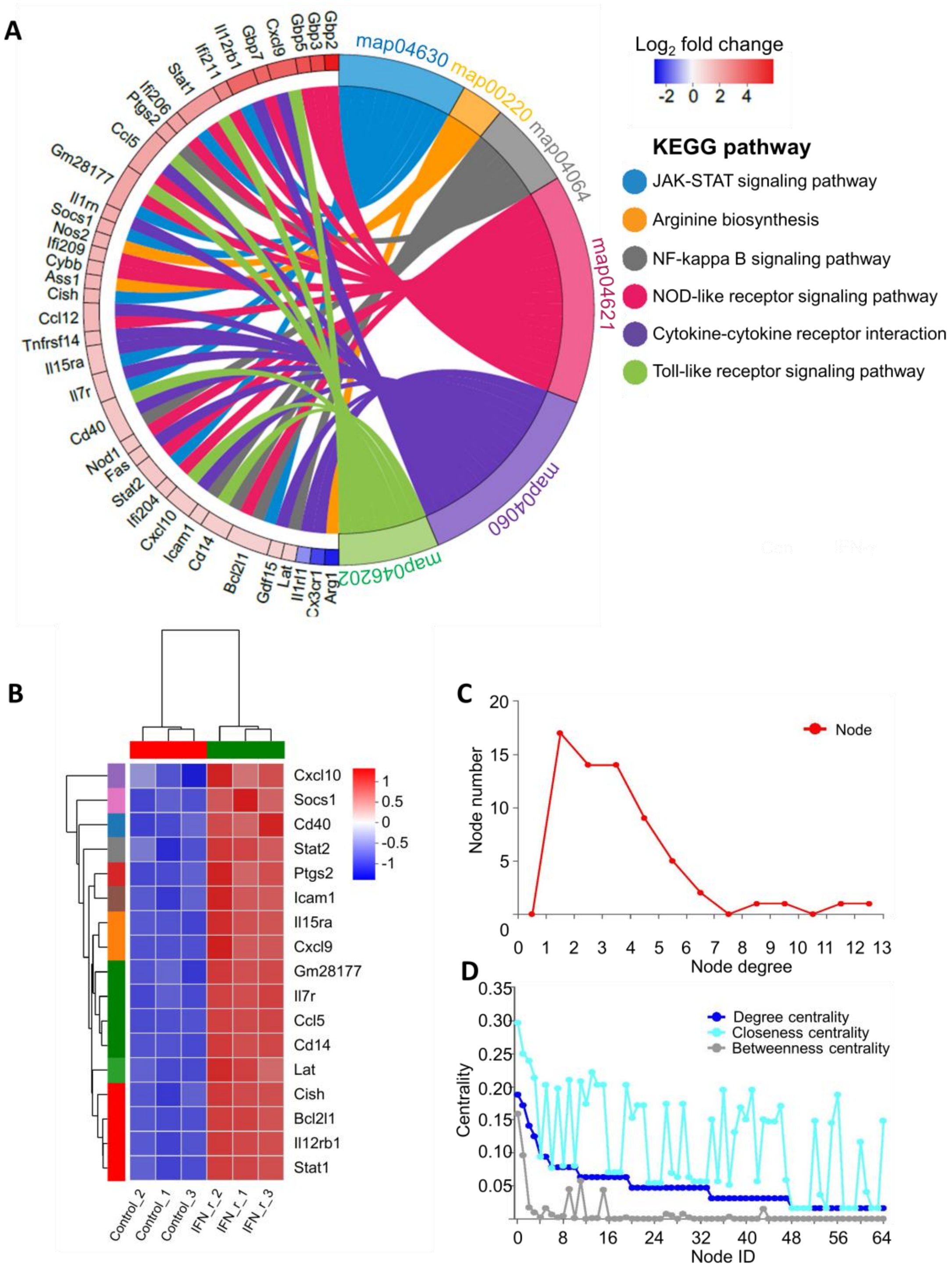

Fig. S2 STAT1 signals were significantly enriched in IFN-γ-primed microglia

(A) Circos plot showing the connectivity map derived from the pairwise comparison of transcriptome datasets. The connectivity between DEGs (left) and Kyoto Encyclopedia of Genes and Genomes (KEGG) term (right) are shown in different color. Each line represents a pairwise dataset overlap, which was determined using Gene Set

Enrichment Analysis and filtered by P < 0.05 and normalized enrichment score > 1.5. The change in the expression of DEGs was quantified as the log2 fold change.

(B) Hierarchical cluster analysis of enriched differentially expressed genes related to Jak-STAT signaling pathway.

(**C** and **D**) Analysis of nodes from interaction network of proteins encoded by differentially expressed genes (DEGs).
