# Supplementary material for: Microglial priming by IFN‐γ involves STAT1‐mediated activation of the NLRP3 inflammasome
Source: CNS Neurosci Ther. 2024 Oct 11;30(10):e70061. doi: 10.1111/cns.70061 (PMC11468839; doi:10.1111/cns.70061)
Supplement: Supplementary file 1 — Data S1. [file CNS-30-e70061-s001.zip › Supplementary Tables.docx]

**Supplementary Tables**

**Supplementary Table 1. Genes primers used for real time PCR analyses**

| **Gene** | **Primer sequences** |
| --- | --- |
| *β-actin* | Forward: 5’-CCGTGAAAAGATGACCCAGATC-3’  Reverse: 5’-CACAGCCTGGATGGCTACGT-3’ |
| *Cd11b* | Forward: 5’- AAACCACAGTCCCGCAGAGA-3’  Reverse: 5’- CGTGTTCACCAGCTGGCTTA-3’ |
| *Cx3cr1* | Forward: 5’-CAGCATCGACCGGTACCTT- 3’  Reverse: 5’-GCTGCACTGTCCGGTTGTT -3’ |
| *Cd200R1* | Forward: 5’-AGGAGGATGAAATGCAGCCTTA-3’  Reverse: 5’- TGCCTCCACCTTAGTCACAGTATC -3’ |
| *Cd86* | Forward: 5’- ATATGACCGTTGTGTGTGTTCTGGA-3’  Reverse: 5’- AGGGCCACAGTAACTGAAGCTGTAA-3’ |
| *NLRP3* | Forward: 5’-ATTACCCGCCCGAGAAAGG-3’  Reverse: 5’-TCGCAGCAAAGATCCACACAG-3’ |
| *Caspase-1* | Forward: 5’-ACAAGGCACGGGACCTATG-3’  Reverse: 5’-TCCCAGTCAGTCCTGGAAATG-3’ |
| *GSDMD* | Forward: 5’- ATGCCATCGGCCTTTGAGAAA-3’  Reverse: 5’- AGGCTGTCCACCGGAATGA-3’ |
| *IL-18* | Forward: 5’- GTGAACCCCAGACCAGACTG-3’  Reverse: 5’- CCTGGAACACGTTTCTGAAAGA -3’ |
| *IL-4* | Forward: 5’-AGGAGGATGAAATGCAGCCTTA-3’  Reverse: 5’- TGCCTCCACCTTAGTCACAGTATC -3’ |
| *TNF-α* | Forward:5’-TACTGAACTTCGGGGTGATTGGTCC-3’  Reverse: 5’-CAGCCTTGTCCCTTGAAGAGAACC-3’ |
| *IL-1β* | Forward: 5’-CCAGCAGGTTATCATCATCATCC-3’  Reverse: 5’-CTCGCAGCAGCACATCAAC-3’ |
| *IL-6* | Forward: 5’- ACCGCTATGAAGTTCCTCTC- 3’  Reverse: 5’- CTCTGTGAAGTCTCCTCTCC-3’ |
| *iNOS* | Forward:5’- ACAACAGGAACCTACCAGCTCA-3’  Reverse: 5’- GATGTTGTAGCGCTGTGTGTCA-3’ |
| *IL-10* | Forward: 5’- TGGCCCAGAAATCAAGGAGC-3’  Reverse: 5’- CAGCAGACTCAATACACACT-3’ |
| *MCR1* | Forward: 5’- CAGCAGACTCAATACACACT-3’  Reverse: 5’- ACTACTACCTGAGCCCACACCTGCT-3’ |
| *Arg-1* | Forward: 5’- AGACAGCAGAGGAGGTGAAGAG- 3’  Reverse: 5’- CGAAGCAAGCCAAGGTTAAAGC-3’ |
| *BDNF* | Forward: 5’-GAGCTGAGCGTGTGTGACAG-3’  Reverse: 5’-CGCCAGCCAATTCTCTTTTTGC-3’ |
| *IGF-1* | Forward: 5’-GGTGGTTTATGAATGGTT-3’  Reverse: 5’-AGGGTGTGTCTAATGGAG-3’ |

**Supplementary Table 2. Primary antibodies used for Western blotting.**

| **Antibody** | **Manufacturer** | **Product numbers** | **Dilution rate** |
| --- | --- | --- | --- |
| GAPDH | Servicebio | GB12002 | 1: 2000 |
| IL-1β | Servicebio | GB11113 | 1: 1000 |
| Caspase1 | Servicebio | GB11383 | 1: 1000 |
| NLRP3 | Affinity | DF7502 | 1: 1000 |
| GSDMD-N | Cell Signaling Technology | 34667 | 1: 1000 |
| GSDMD | ThermoFisher Scientific | PA5-116815 | 1:1000 |
| pro-IL-1β | Cell Signaling Technology | 12242 | 1:1000 |
| Pro-caspase-1 | Abcam | Ab179515 | 1:1000 |
| p-STAT1(Tyr701) | Cell Signaling Technology | 9167S | 1:400 |
| STAT1 | Cell Signaling Technology | 14994S | 1:800 |

**Table S3. Primary antibodies used for immunofluorescence.**

| **Antibody** | **Manufacturer** | **Product number** | **Dilution** |
| --- | --- | --- | --- |
| Iba1 | Abcam | Ab178846 | 1:400 |
| GFAP | Cell Signaling Technology | 80788S | 1:400 |
| NeuN | Cell Signaling Technology | 24307S | 1:100 |
| CX3CR1 | ThermoFisher Scientific | 702321 | 1:250 |
| NG2 | Abcam | ab275024 | 1:400 |
| MAP2 | Cell Signaling Technology | 8707S | 1:400 |
| Cleaved-caspase 3 | Cell Signaling Technology | 9661S | 1:300 |
| iNOS | Abcam | ab49999 | 1:400 |
| GSDMD | Thermo Fisher Scientific | PA5-116815 | 1:200 |
| NLRP3 | Abcam | ab272702 | 1:300 |
| IL-1β | Cell Signaling Technology | 31202S | 1:200 |
| Caspase 1 | ThermoFisher Scientific | MA5-16215 | 1:300 |

**Table S4．The F value and P value in multiple comparisons of figure 1**

| **Item** | **Marker** | **Groups** | **t, df** | **Type of Test** | **95% confidence interval** | **F, DFn, Dfd** | **P value** |
| --- | --- | --- | --- | --- | --- | --- | --- |
| *mRNA expression* | *CD68* | CON vs. IFN-γ | t=5.216, df=6 | Unpaired t test | 1.042 to 2.883 | 6.287, 3, 3 | 0.0020 |
|  | *CD11b* |  | t=7.079, df=6 |  | 1.395 to 2.870 | 1.859, 3, 3 | 0.0004 |
|  | *CX3CR1* |  | t=4.980, df=6 |  | -1.029 to -0.3510 | 2.751, 3, 3 | 0.0025 |
|  | *CD200* |  | t=4.092, df=6 |  | -1.067 to -0.2683 | 7.344, 3, 3 | 0.0064 |
|  | *IL-6* |  | t=3.919, df=6 |  | 0.4658 to 2.014 | 1.614, 3, 3 | 0.0078 |
|  | *IL-1β* |  | t=6.130, df=6 |  | 1.658 to 3.862 | 2.001, 3, 3 | 0.0009 |
|  | *TNF-α* |  | t=2.715, df=6 |  | 0.08533 to 1.645 | 2.786, 3, 3 | 0.0349 |
|  | *iNOS* |  | t=6.269, df=6 |  | 1.480 to 3.375 | 1.260, 3, 3 | 0.0008 |
|  | *IL-10* |  | t=1.829, df=6 |  | -0.8007 to 0.1157 | 2.191, 3, 3 | 0.1171 |
|  | *IL-4* |  | t=2.383, df=6 |  | -0.6891 to 0.009099 | 1.335, 3, 3 | 0.0545 |
|  | *Arg1* |  | t=3.550, df=6 |  | -0.9967 to -0.1833 | 5.049, 3, 3 | 0.0121 |
|  | *MCR1* |  | t=2.799, df=6 |  | -1.307 to -0.08778 | 13.70, 3, 3 | 0.0312 |
|  | *BDNF* |  | t=4.816, df=6 |  | -0.8596 to -0.2804 | 1.117, 3, 3 | 0.0030 |
|  | *IGF-1* |  | t=6.076, df=6 |  | -0.9503 to -0.4047 | 1.223, 3, 3 | 0.0009 |
| *Meanfluorescence*  *intensity × 10^6^ (AU)* | *CX3CR1* |  | t=3.749, df=6 |  | -2.023 to -0.4251 | 4.135, 3, 3 | 0.0095 |
|  | *iNOS* |  | t=8.695, df=6 |  | 3.033 to 5.408 | 33.06, 3, 3 | 0.0001 |
|  | *NLRP3* |  | t=4.645, df=6 |  | 0.9588 to 3.094 | 1.826, 3, 3 | 0.0035 |
|  | *caspase-1* |  | t=5.996, df=6 |  | 0.7171 to 1.706 | 9.253, 3, 3 | 0.0010 |
|  | *GSDMD* |  | t=4.645, df=6 |  | 0.6585 to 2.124 | 17.06, 3, 3 | 0.0035 |
|  | *IL-1β* |  | t=4.628, df=6 |  | 0.3567 to 1.157 | 8.425, 3, 3 | 0.0036 |

**Table S5．The F value and P value in multiple comparisons of figure 2**

| **Marker** | **Groups** | **Mean Diff.** | **Type of ANOVA** | **95.00% CI of diff.** | **F, DFn, Dfd** | **P value** |
| --- | --- | --- | --- | --- | --- | --- |
| *Proportion of microglia types 1* | Ctrl vs. IFN-γ | 61.79 | One-Way ANOVA  Tukey’s post hoc tests | 55.35 to 68.23 | F (3, 12) = 446.6 | <0.0001 |
|  | Ctrl vs. LPS | 64.81 |  | 58.37 to 71.25 |  | <0.0001 |
|  | Ctrl vs. IFN-γ+LPS | 67.46 |  | 61.01 to 73.90 |  | <0.0001 |
| *Proportion of microglia types 2* | Ctrl vs. IFN-γ | -50.63 | One-Way ANOVA  Tukey’s post hoc tests | -61.97 to -39.29 | F (3, 12) = 108.9 | <0.0001 |
|  | IFN-γ vs. LPS | 53.53 |  | 42.19 to 64.86 |  | <0.0001 |
|  | IFN-γ vs. IFN-γ+LPS | 62.27 |  | 50.94 to 73.61 |  | <0.0001 |
| *Proportion of microglia types 3* | Ctrl vs. LPS | -67.70 | One-Way ANOVA  Tukey’s post hoc tests | -79.05 to -56.36 | F (3, 12) = 215.8 | <0.0001 |
|  | Ctrl vs. IFN-γ+LPS | 79.10 |  | -90.44 to -67.75 |  | <0.0001 |
|  | IFN-γ vs. LPS | -56.55 |  | -67.89 to -45.20 |  | <0.0001 |
|  | FN-γ vs. IFN-γ+LPS | -67.94 |  | -79.29 to -56.59 |  | <0.0001 |
| *Perimeter of microglia (μm)* | Con vs. IFN-γ | -12.13 | One-Way ANOVA  Tukey’s post hoc tests | -16.58 to -7.684 | F (3, 333) = 26.82 | <0.0001 |
|  | Con vs. LPS | -14.15 |  | -18.60 to -9.707 |  | <0.0001 |
|  | Con vs. IFN-γ+LPS | -7.082 |  | -11.52 to -2.647 |  | 0.0003 |
|  | IFN-γ vs. IFN-γ+LPS | 5.049 |  | 0.6150 to 9.483 |  | 0.0183 |
|  | LPS vs. IFN-γ+LPS | 7.073 |  | 2.639 to 11.51 |  | 0.0003 |
| *Area of microglia (μm2)* | Con vs. IFN-γ | -122.3 | One-Way ANOVA  Tukey’s post hoc tests | -155.5 to -89.01 | F (3, 407) = 70.29 | <0.0001 |
|  | Con vs. LPS | -183.7 |  | -217.0 to -150.5 |  | <0.0001 |
|  | Con vs. IFN-γ+LPS | -95.29 |  | -128.6 to -61.94 |  | <0.0001 |
|  | IFN-γ vs. LPS | -61.47 |  | -94.74 to -28.21 |  | <0.0001 |
|  | IFN-γ vs. IFN-γ+LPS | 88.46 |  | 55.11 to 121.8 |  | <0.0001 |
| *Microbeads phagocytosed by each cell* | Con vs. IFN-γ | -1.922 | One-Way ANOVA  Tukey’s post hoc tests | -3.356 to -0.4884 | F (3, 458) = 81.58 | 0.0033 |
|  | Con vs. LPS | -4.046 |  | -5.493 to -2.600 |  | <0.0001 |
|  | Con vs. IFN-γ+LPS | -8.182 |  | -9.601 to -6.764 |  | <0.0001 |
|  | IFN-γ vs. LPS | -2.124 |  | -3.574 to -0.6747 |  | 0.0010 |
|  | IFN-γ vs. IFN-γ+LPS | -6.261 |  | -7.682 to -4.839 |  | <0.0001 |
|  | LPS vs. IFN-γ+LPS | -4.136 |  | -5.571 to -2.702 |  | <0.0001 |
| *mRNA expression NLRP3* | Con vs. IFN-γ | -1.823 | One-Way ANOVA  Tukey’s post hoc tests | -3.443 to -0.2022 | F (3, 12) = 42.10 | 0.0262 |
|  | Con vs. LPS | -2.820 |  | -4.440 to -1.200 |  | 0.0012 |
|  | Con vs. IFN-γ+LPS | -5.978 |  | -7.598 to -4.357 |  | <0.0001 |
|  | IFN-γ vs. IFN-γ+LPS | -4.155 |  | -5.775 to -2.535 |  | <0.0001 |
|  | LPS vs. IFN-γ+LPS | -3.158 |  | -4.778 to -1.537 |  | 0.0004 |
| *mRNA expression Casepase-1* | Con vs. IFN-γ | -1.698 | One-Way ANOVA  Tukey’s post hoc tests | -2.764 to -0.6311 | F (3, 12) = 42.95 | 0.0024 |
|  | Con vs. LPS | -2.570 |  | -3.636 to -1.504 |  | <0.0001 |
|  | Con vs. IFN-γ+LPS | -3.978 |  | -5.044 to -2.911 |  | <0.0001 |
|  | IFN-γ vs. IFN-γ+LPS | -2.280 |  | -3.346 to -1.214 |  | 0.0002 |
|  | LPS vs. IFN-γ+LPS | -1.408 |  | -2.474 to -0.3411 |  | 0.0095 |
| *mRNA expression GSDMD* | Con vs. IFN-γ | -1.315 | One-Way ANOVA  Tukey’s post hoc tests | -2.490 to -0.1403 | F (3, 12) = 19.97 | 0.0270 |
|  | Con vs. LPS | -1.993 |  | -3.167 to -0.8178 |  | 0.0014 |
|  | Con vs. IFN-γ+LPS | -2.978 |  | -4.152 to -1.803 |  | <0.0001 |
|  | IFN-γ vs. IFN-γ+LPS | -1.663 |  | -2.837 to -0.4878 |  | 0.0058 |
| *mRNA expression IL-1β* | Con vs. IFN-γ | -4.390 | One-Way ANOVA  Tukey’s post hoc tests | -8.293 to -0.4866 | F (3, 12) = 54.37 | 0.0262 |
|  | Con vs. LPS | -6.570 |  | -10.47 to -2.667 |  | 0.0015 |
|  | Con vs. IFN-γ+LPS | -16.23 |  | -20.13 to -12.32 |  | <0.0001 |
|  | IFN-γ vs. IFN-γ+LPS | -11.84 |  | -15.74 to -7.934 |  | <0.0001 |
|  | LPS vs. IFN-γ+LPS | -9.658 |  | -13.56 to -5.754 |  | <0.0001 |
| *mRNA expression IL-18* | Con vs. LPS | -5.070 | One-Way ANOVA  Tukey’s post hoc tests | -7.712 to -2.428 | F (3, 12) = 38.43 | 0.0005 |
|  | Con vs. IFN-γ+LPS | -8.978 |  | -11.62 to -6.335 |  | <0.0001 |
|  | IFN-γ vs. LPS | -3.005 |  | -5.647 to -0.3625 |  | 0.0246 |
|  | IFN-γ vs. IFN-γ+LPS | -6.913 |  | -9.555 to -4.270 |  | <0.0001 |
|  | LPS vs. IFN-γ+LPS | -3.908 |  | -6.550 to -1.265 |  | 0.0042 |
| *mRNA expression TNF-α* | Con vs. LPS | -3.570 | One-Way ANOVA  Tukey’s post hoc tests | -5.928 to -1.212 | F (3, 12) = 36.62 | 0.0035 |
|  | Con vs. IFN-γ+LPS | -7.478 |  | -9.836 to -5.119 |  | <0.0001 |
|  | IFN-γ vs. LPS | -2.873 |  | -5.231 to -0.5143 |  | 0.0161 |
|  | IFN-γ vs. IFN-γ+LPS | -6.780 |  | -9.138 to -4.422 |  | <0.0001 |
|  | LPS vs. IFN-γ+LPS | -3.908 |  | -6.266 to -1.549 |  | 0.0017 |
| *mRNA expression iNOS* | Con vs. IFN-γ | -4.698 | One-Way ANOVA  Tukey’s post hoc tests | -7.708 to -1.687 | F (3, 12) = 52.22 | 0.0028 |
|  | Con vs. LPS | -8.070 |  | -11.08 to -5.060 |  | <0.0001 |
|  | Con vs. IFN-γ+LPS | -12.23 |  | -15.24 to -9.217 |  | <0.0001 |
|  | IFN-γ vs. LPS | -3.373 |  | -6.383 to -0.3623 |  | 0.0268 |
|  | IFN-γ vs. IFN-γ+LPS | -7.530 |  | -10.54 to -4.520 |  | <0.0001 |
|  | LPS vs. IFN-γ+LPS | -4.158 |  | -7.168 to -1.147 |  | 0.0069 |
| *Nitric oxide concentration*  *in medium (μM)* | Con vs. IFN-γ | -2.307 | One-Way ANOVA  Tukey’s post hoc tests | -3.585 to -1.028 | F (3, 12) = 32.52 | 0.0009 |
|  | Con vs. LPS | -2.602 |  | -3.880 to -1.323 |  | 0.0003 |
|  | Con vs. IFN-γ+LPS | -4.214 |  | -5.492 to -2.936 |  | <0.0001 |
|  | IFN-γ vs. IFN-γ+LPS | -1.908 |  | -3.186 to -0.6292 |  | 0.0039 |
|  | LPS vs. IFN-γ+LPS | -1.613 |  | -2.891 to -0.3342 |  | 0.0129 |
| *Protein level*  *NLRP3* | Con vs. IFN-γ | -0.7367 | One-Way ANOVA  Tukey’s post hoc tests | -1.010 to -0.4634 | F (3, 8) = 102.5 | 0.0001 |
|  | Con vs. LPS | -0.8627 |  | -1.136 to -0.5894 |  | <0.0001 |
|  | Con vs. IFN-γ+LPS | -1.490 |  | -1.763 to -1.216 |  | <0.0001 |
|  | IFN-γ vs. IFN-γ+LPS | -0.7530 |  | -1.026 to -0.4797 |  | <0.0001 |
|  | LPS vs. IFN-γ+LPS | -0.6270 |  | -0.9003 to -0.3537 |  | 0.0004 |
| *Protein level*  *Pro-caspase-1* | Con vs. IFN-γ | -0.2660 | One-Way ANOVA  Tukey’s post hoc tests | -0.4753 to -0.05663 | F (3, 8) = 38.50 | 0.0152 |
|  | Con vs. LPS | -0.2582 |  | -0.4675 to -0.04886 |  | 0.0178 |
|  | Con vs. IFN-γ+LPS | -0.6923 |  | -0.9016 to -0.4830 |  | <0.0001 |
|  | IFN-γ vs. IFN-γ+LPS | -0.4264 |  | -0.6357 to -0.2170 |  | 0.0008 |
|  | LPS vs. IFN-γ+LPS | -0.4341 |  | -0.6435 to -0.2248 |  | 0.0007 |
| *Protein level*  *Pro-IL-1β* | Con vs. IFN-γ | -0.9438 | One-Way ANOVA  Tukey’s post hoc tests | -1.108 to -0.7799 | F (3, 8) = 252.9 | <0.0001 |
|  | Con vs. LPS | -0.8862 |  | -1.050 to -0.7223 |  | <0.0001 |
|  | Con vs. IFN-γ+LPS | -1.371 |  | -1.535 to -1.207 |  | <0.0001 |
|  | IFN-γ vs. IFN-γ+LPS | -0.4269 |  | -0.5908 to -0.2629 |  | 0.0001 |
|  | LPS vs. IFN-γ+LPS | -0.4845 |  | -0.6484 to -0.3206 |  | <0.0001 |
| *Protein level*  *GSDMD-FL* | Con vs. IFN-γ | -0.1412 | One-Way ANOVA  Tukey’s post hoc tests | -0.2682 to -0.01431 | F (3, 8) = 60.85 | 0.0302 |
|  | Con vs. LPS | -0.2533 |  | -0.3802 to -0.1263 |  | 0.0010 |
|  | Con vs. IFN-γ+LPS | -0.5166 |  | -0.6435 to -0.3897 |  | <0.0001 |
|  | IFN-γ vs. IFN-γ+LPS | -0.3753 |  | -0.5023 to -0.2484 |  | <0.0001 |
|  | LPS vs. IFN-γ+LPS | -0.2633 |  | -0.3903 to -0.1364 |  | 0.0007 |
| *Protein level*  *Caspase-1 / Pro-caspase-1* | Con vs. IFN-γ | -0.2175 | One-Way ANOVA  Tukey’s post hoc tests | -0.2650 to -0.1700 | F (3, 8) = 627.2 | <0.0001 |
|  | Con vs. LPS | -0.2407 |  | -0.2882 to -0.1933 |  | <0.0001 |
|  | Con vs. IFN-γ+LPS | -0.6309 |  | -0.6784 to -0.5834 |  | <0.0001 |
|  | IFN-γ vs. IFN-γ+LPS | -0.4134 |  | -0.4609 to -0.3660 |  | <0.0001 |
|  | LPS vs. IFN-γ+LPS | -0.3902 |  | -0.4376 to -0.3427 |  | <0.0001 |
| *Protein level*  *GSDMD-N / GSDMD-FL* | Con vs. IFN-γ | -0.1628 | One-Way ANOVA  Tukey’s post hoc tests | -0.2952 to -0.03029 | F (3, 8) = 226.4 | 0.0182 |
|  | Con vs. LPS | -0.3280 |  | -0.4605 to -0.1956 |  | 0.0002 |
|  | Con vs. IFN-γ+LPS | -1.002 |  | -1.135 to -0.8696 |  | <0.0001 |
|  | IFN-γ vs. LPS | -0.1653 |  | -0.2977 to -0.03280 |  | 0.0168 |
|  | IFN-γ vs. IFN-γ+LPS | -0.8393 |  | -0.9718 to -0.7069 |  | <0.0001 |
|  | LPS vs. IFN-γ+LPS | -0.6741 |  | -0.8065 to -0.5416 |  | <0.0001 |
| *Protein level*  *IL-1β* | Con vs. IFN-γ | -0.4040 | One-Way ANOVA  Tukey’s post hoc tests | -0.5036 to -0.3043 | F (3, 8) = 228.1 | <0.0001 |
|  | Con vs. LPS | -0.3193 |  | -0.4190 to -0.2196 |  | <0.0001 |
|  | Con vs. IFN-γ+LPS | -0.8074 |  | -0.9071 to -0.7078 |  | <0.0001 |
|  | IFN-γ vs. IFN-γ+LPS | -0.4035 |  | -0.5031 to -0.3038 |  | <0.0001 |
|  | LPS vs. IFN-γ+LPS | -0.4881 |  | -0.5878 to -0.3885 |  | <0.0001 |
| *IL-1β in medium (pg/ml)* | Con vs. IFN-γ | -21.37 | One-Way ANOVA  Tukey’s post hoc tests | -38.32 to -4.417 | F (3, 8) = 26.16 | 0.0159 |
|  | Con vs. LPS | -26.66 |  | -43.61 to -9.707 |  | 0.0044 |
|  | Con vs. IFN-γ+LPS | -46.58 |  | -63.53 to -29.63 |  | 0.0001 |
|  | IFN-γ vs. IFN-γ+LPS | -25.21 |  | -42.16 to -8.260 |  | 0.0062 |
|  | LPS vs. IFN-γ+LPS | -19.92 |  | -36.87 to -2.970 |  | 0.0229 |

**Table S6．The F value and P value in multiple comparisons of figure 3**

| **Marker** | **Groups** | **Mean Diff.** | **Type of ANOVA** | **95.00% CI of diff.** | **F, DFn, Dfd** | **P value** |
| --- | --- | --- | --- | --- | --- | --- |
| *Cleaved caspase 3^+^ cells (%)* | Con vs. IFN-γ | -2.917 | One-Way ANOVA  Tukey’s post hoc tests | -5.323 to -0.5108 | F (3, 12) = 61.17 | 0.0166 |
|  | Con vs. LPS | -4.467 |  | -6.873 to -2.061 |  | 0.0007 |
|  | Con vs. IFN-γ+LPS | -10.62 |  | -13.03 to -8.218 |  | <0.0001 |
|  | IFN-γ vs. IFN-γ+LPS | -7.708 |  | -10.11 to -5.301 |  | <0.0001 |
|  | LPS vs. IFN-γ+LPS | -6.158 |  | -8.564 to -3.751 |  | <0.0001 |
| *MAP2+ cells (%)* | Con vs. IFN-γ | 4.117 | One-Way ANOVA  Tukey’s post hoc tests | 1.861 to 6.372 | F (3, 12) = 64.84 | 0.0008 |
|  | Con vs. LPS | 7.093 |  | 4.838 to 9.348 |  | <0.0001 |
|  | Con vs. IFN-γ+LPS | 10.14 |  | 7.885 to 12.40 |  | <0.0001 |
|  | IFN-γ vs. LPS | 2.977 |  | 0.7210 to 5.232 |  | 0.0095 |
|  | IFN-γ vs. IFN-γ+LPS | 6.024 |  | 3.769 to 8.280 |  | <0.0001 |
|  | LPS vs. IFN-γ+LPS | 3.048 |  | 0.7923 to 5.303 |  | 0.0081 |
| *NG2+ cells (%)* | Con vs. IFN-γ | 5.383 | One-Way ANOVA  Tukey’s post hoc tests | 1.788 to 8.978 | F (3, 12) = 29.82 | 0.0038 |
|  | Con vs. LPS | 6.556 |  | 2.961 to 10.15 |  | 0.0008 |
|  | Con vs. IFN-γ+LPS | 11.39 |  | 7.792 to 14.98 |  | <0.0001 |
|  | IFN-γ vs. IFN-γ+LPS | 6.004 |  | 2.409 to 9.599 |  | 0.0016 |
|  | LPS vs. IFN-γ+LPS | 4.832 |  | 1.236 to 8.427 |  | 0.0084 |
| *GFAP+ cells (%)* | Con vs. LPS | -11.68 | One-Way ANOVA  Tukey’s post hoc tests | -20.84 to -2.525 | F (3, 12) = 9.872 | 0.0120 |
|  | Con vs. IFN-γ+LPS | -15.90 |  | -25.06 to -6.747 |  | 0.0012 |
|  | IFN-γ vs. IFN-γ+LPS | -9.321 |  | -18.48 to -0.1640 |  | 0.0456 |

**Table S7．The F value and P value in multiple comparisons of figure 4**

| **Marker** | **Groups** | **Mean Diff.** | **Type of ANOVA** | **95.00% CI of diff.** | **F, DFn, Dfd** | **P value** |
| --- | --- | --- | --- | --- | --- | --- |
| *Protein level*  *STAT1* | Con vs. IFN-γ | -0.5436 | One-Way ANOVA  Tukey’s post hoc tests | -0.6384 to -0.4488 | F (3, 8) = 177.4 | <0.0001 |
|  | Con vs. LPS | -0.3266 |  | -0.4214 to -0.2318 |  | <0.0001 |
|  | Con vs. IFN-γ+LPS | -0.6236 |  | -0.7184 to -0.5288 |  | <0.0001 |
|  | IFN-γ vs. LPS | 0.2170 |  | 0.1222 to 0.3118 |  | 0.0004 |
|  | LPS vs. IFN-γ+LPS | -0.2970 |  | -0.3918 to -0.2022 |  | <0.0001 |
| *Protein level*  *p-STAT1* | Con vs. IFN-γ | -0.2246 | One-Way ANOVA  Tukey’s post hoc tests | -0.2719 to -0.1774 | F (3, 8) = 368.0 | <0.0001 |
|  | Con vs. LPS | -0.1098 |  | -0.1570 to -0.06252 |  | 0.0003 |
|  | Con vs. IFN-γ+LPS | -0.4673 |  | -0.5146 to -0.4200 |  | <0.0001 |
|  | IFN-γ vs. LPS | 0.1149 |  | 0.06760 to 0.1621 |  | 0.0002 |
|  | IFN-γ vs. IFN-γ+LPS | -0.2427 |  | -0.2899 to -0.1954 |  | <0.0001 |
|  | LPS vs. IFN-γ+LPS | -0.3575 |  | -0.4048 to -0.3103 |  | <0.0001 |
| *Mean fluorescence intensity of total STAT1*  *× 10^6^ (AU)* | Con vs. IFN-γ | -0.9698 | One-Way ANOVA  Tukey’s post hoc tests | -1.263 to -0.6765 | F (5, 18) = 72.50 | <0.0001 |
|  | IFN-γ vs. IFN-γ+fludarabin | 0.8006 |  | 0.5074 to 1.094 |  | <0.0001 |
|  | LPS vs. IFN-γ+LPS | -1.004 |  | -1.297 to -0.7109 |  | <0.0001 |
|  | IFN-γ+LPS vs. IFN-γ+LPS+fludarabin | 1.115 |  | 0.8221 to 1.409 |  | <0.0001 |
| *Mean fluorescence*  *intensity of endonuclear*  *STAT1 × 10^5^ (AU)* | Con vs. IFN-γ | -1.946 | One-Way ANOVA  Tukey’s post hoc tests | -2.665 to -1.228 | F (5, 18) = 35.37 | <0.0001 |
|  | IFN-γ vs. IFN-γ+fludarabin | 1.882 |  | 1.163 to 2.600 |  | <0.0001 |
|  | LPS vs. IFN-γ+LPS | -1.238 |  | -1.956 to -0.5193 |  | 0.0004 |
|  | IFN-γ+LPS vs. IFN-γ+LPS+fludarabin | 1.877 |  | 1.159 to 2.596 |  | <0.0001 |
| *Mean fluorescence*  *intensity of NLRP3*  *× 10^6^ (AU)* | Con vs. IFN-γ | -2.229 | One-Way ANOVA  Tukey’s post hoc tests | -2.545 to -1.913 | F (5, 18) = 282.5 | <0.0001 |
|  | IFN-γ vs. IFN-γ+fludarabin | 1.706 |  | 1.390 to 2.022 |  | <0.0001 |
|  | LPS vs. IFN-γ+LPS | -2.430 |  | -2.746 to -2.114 |  | <0.0001 |
|  | IFN-γ+LPS vs. IFN-γ+LPS+fludarabin | 1.518 |  | 1.202 to 1.834 |  | <0.0001 |
| *mRNA expression*  *NLRP3* | Con vs. IFN-γ | -1.670 | One-Way ANOVA  Tukey’s post hoc tests | -2.573 to -0.7663 | F (5, 18) = 40.37 | 0.0002 |
|  | IFN-γ vs. IFN-γ+fludarabin | 1.464 |  | 0.5601 to 2.367 |  | 0.0008 |
|  | LPS vs. IFN-γ+LPS | -1.544 |  | -2.448 to -0.6404 |  | 0.0005 |
|  | IFN-γ+LPS vs. IFN-γ+LPS+fludarabin | 2.224 |  | 1.320 to 3.127 |  | <0.0001 |
| *mRNA expression*  *Caspase-1* | Con vs. IFN-γ | -1.759 | One-Way ANOVA  Tukey’s post hoc tests | -2.933 to -0.5857 | F (5, 18) = 30.69 | 0.0018 |
|  | IFN-γ vs. IFN-γ+fludarabin | 1.384 |  | 0.2100 to 2.557 |  | 0.0157 |
|  | LPS vs. IFN-γ+LPS | -1.383 |  | -2.556 to -0.2090 |  | 0.0158 |
|  | IFN-γ+LPS vs. IFN-γ+LPS+fludarabin | 2.805 |  | 1.631 to 3.978 |  | <0.0001 |
| *mRNA expression*  *GSDMD* | Con vs. IFN-γ | -1.724 | One-Way ANOVA  Tukey’s post hoc tests | -2.709 to -0.7385 | F (5, 18) = 33.11 | 0.0003 |
|  | IFN-γ vs. IFN-γ+fludarabin | 1.645 |  | 0.6595 to 2.630 |  | 0.0006 |
|  | LPS vs. IFN-γ+LPS | -2.115 |  | -3.100 to -1.129 |  | <0.0001 |
|  | IFN-γ+LPS vs. IFN-γ+LPS+fludarabin | 2.167 |  | 1.182 to 3.153 |  | <0.0001 |
| *mRNA expression*  *IL-1β* | Con vs. IFN-γ | -4.968 | One-Way ANOVA  Tukey’s post hoc tests | -8.436 to -1.500 | F (5, 18) = 60.88 | 0.0029 |
|  | IFN-γ vs. IFN-γ+fludarabin | 3.923 |  | 0.4554 to 7.391 |  | 0.0215 |
|  | LPS vs. IFN-γ+LPS | -11.67 |  | -15.14 to -8.204 |  | <0.0001 |
|  | IFN-γ+LPS vs. IFN-γ+LPS+fludarabin | 9.692 |  | 6.224 to 13.16 |  | <0.0001 |
| *mRNA expression*  *IL-18* | Con vs. IFN-γ | -2.313 | One-Way ANOVA  Tukey’s post hoc tests | -4.100 to -0.5265 | F (5, 18) = 54.02 | 0.0072 |
|  | IFN-γ vs. IFN-γ+fludarabin | 1.925 |  | 0.1383 to 3.712 |  | 0.0305 |
|  | LPS vs. IFN-γ+LPS | -4.696 |  | -6.483 to -2.909 |  | <0.0001 |
|  | IFN-γ+LPS vs. IFN-γ+LPS+fludarabin | 5.916 |  | 4.129 to 7.703 |  | <0.0001 |
| *mRNA expression*  *iNOS* | Con vs. IFN-γ | -3.461 | One-Way ANOVA  Tukey’s post hoc tests | -5.456 to -1.467 | F (5, 18) = 48.42 | 0.0004 |
|  | IFN-γ vs. IFN-γ+fludarabin | 2.791 |  | 0.7961 to 4.786 |  | 0.0036 |
|  | LPS vs. IFN-γ+LPS | -4.636 |  | -6.631 to -2.641 |  | <0.0001 |
|  | IFN-γ+LPS vs. IFN-γ+LPS+fludarabin | 5.826 |  | 3.832 to 7.821 |  | <0.0001 |
| *mRNA expression*  *TNF-α* | Con vs. LPS | -2.763 | One-Way ANOVA  Tukey’s post hoc tests | -3.870 to -1.656 | F (5, 18) = 70.51 | <0.0001 |
|  | LPS vs. IFN-γ+LPS | -2.573 |  | -3.680 to -1.466 |  | <0.0001 |
|  | IFN-γ+LPS vs. IFN-γ+LPS+fludarabin | 3.288 |  | 2.181 to 4.395 |  | <0.0001 |
| *Nitric oxide*  *in medium (μM)* | Con vs. IFN-γ | -2.355 | One-Way ANOVA  Tukey’s post hoc tests | -3.526 to -1.184 | F (5, 18) = 45.11 | <0.0001 |
|  | IFN-γ vs. IFN-γ+fludarabin | 1.958 |  | 0.7864 to 3.129 |  | 0.0006 |
|  | LPS vs. IFN-γ+LPS | -2.064 |  | -3.235 to -0.8924 |  | 0.0003 |
|  | IFN-γ+LPS vs. IFN-γ+LPS+fludarabin | 2.907 |  | 1.735 to 4.078 |  | <0.0001 |
| *IL-1β in medium*  *(pg/ml)* | Con vs. IFN-γ | -13.87 | One-Way ANOVA  Tukey’s post hoc tests | -24.69 to -3.044 | F (5, 18) = 44.12 | 0.0079 |
|  | IFN-γ vs. IFN-γ+fludarabin | 12.43 |  | 1.601 to 23.25 |  | 0.0193 |
|  | LPS vs. IFN-γ+LPS | -24.29 |  | -35.11 to -13.47 |  | <0.0001 |
|  | IFN-γ+LPS vs. IFN-γ+LPS+fludarabin | 25.32 |  | 14.50 to 36.15 |  | <0.0001 |

**Table S8．The F value and P value in multiple comparisons of figure 5**

| **Marker** | **Groups** | **Mean Diff.** | **Type of ANOVA** | **95.00% CI of diff.** | **F, DFn, Dfd** | **P value** |
| --- | --- | --- | --- | --- | --- | --- |
| *Mean fluorescence intensity*  *of NLRP3 × 10^6^ (AU)* | Con vs. IFN-γ | -4.175 | One-Way ANOVA  Tukey’s post hoc tests | -5.554 to -2.795 | F (5, 18) = 134.1 | <0.0001 |
|  | IFN-γ vs. IFN-γ+LPS | -3.950 |  | -5.330 to -2.571 |  | <0.0001 |
|  | IFN-γ vs. IFN-γ+fludarabin | 3.865 |  | 2.486 to 5.245 |  | <0.0001 |
|  | IFN-γ+LPS vs. IFN-γ+LPS+fludarabin | 5.131 |  | 3.752 to 6.511 |  | <0.0001 |
| *Mean fluorescence intensity*  *of Caspase-1 × 10^6^ (AU)* | Con vs. IFN-γ | -1.105 | One-Way ANOVA  Tukey’s post hoc tests | -1.809 to -0.4001 | F (5, 18) = 61.86 | 0.0012 |
|  | IFN-γ vs. IFN-γ+fludarabin | 0.8756 |  | 0.1710 to 1.580 |  | 0.0103 |
|  | LPS vs. IFN-γ+LPS | -1.238 |  | -1.943 to -0.5336 |  | 0.0003 |
|  | IFN-γ+LPS vs. IFN-γ+LPS+fludarabin | 3.058 |  | 2.353 to 3.763 |  | <0.0001 |
| *Mean fluorescence intensity*  *of GSDMD × 10^6^ (AU)* | Con vs. IFN-γ | -2.947 | One-Way ANOVA  Tukey’s post hoc tests | -4.100 to -1.794 | F (5, 18) = 57.65 | <0.0001 |
|  | IFN-γ vs. IFN-γ+fludarabin | 2.702 |  | 1.549 to 3.855 |  | <0.0001 |
|  | LPS vs. IFN-γ+LPS | -1.834 |  | -2.987 to -0.6816 |  | 0.0010 |
|  | IFN-γ+LPS vs. IFN-γ+LPS+fludarabin | 2.982 |  | 1.830 to 4.135 |  | <0.0001 |
| *Mean fluorescence*  *intensity of CX3CR1*  *× 10^6^ (AU)* | Con vs. IFN-γ | 3.296 | One-Way ANOVA  Tukey’s post hoc tests | 1.647 to 4.945 | F (5, 18) = 20.18 | <0.0001 |
|  | IFN-γ vs. IFN-γ+fludarabin | -2.123 |  | -3.772 to -0.4740 |  | 0.0076 |
|  | LPS vs. IFN-γ+LPS | -2.068 |  | -3.717 to -0.4184 |  | 0.0095 |
|  | IFN-γ+LPS vs. IFN-γ+LPS+fludarabin | -1.929 |  | -3.579 to -0.2802 |  | 0.0167 |
| *Mean fluorescence*  *intensity of iNOS*  *× 10^5^ (AU)* | Con vs. IFN-γ | -6.798 | One-Way ANOVA  Tukey’s post hoc tests | -8.760 to -4.837 | F (5, 18) = 129.3 | <0.0001 |
|  | IFN-γ vs. IFN-γ+fludarabin | 5.218 |  | 3.257 to 7.179 |  | <0.0001 |
|  | LPS vs. IFN-γ+LPS | -2.948 |  | -4.909 to -0.9865 |  | 0.0018 |
|  | IFN-γ+LPS vs. IFN-γ+LPS+fludarabin | 10.36 |  | 8.400 to 12.32 |  | <0.0001 |
| *Nitric oxide*  *in medium (μM)* | Con vs. IFN-γ | -2.831 | One-Way ANOVA  Tukey’s post hoc tests | -4.314 to -1.347 | F (5, 18) = 39.67 | 0.0001 |
|  | IFN-γ vs. IFN-γ+fludarabin | 2.228 |  | 0.7450 to 3.712 |  | 0.0018 |
|  | LPS vs. IFN-γ+LPS | -2.238 |  | -3.721 to -0.7544 |  | 0.0017 |
|  | IFN-γ+LPS vs. IFN-γ+LPS+fludarabin | 3.377 |  | 1.894 to 4.861 |  | <0.0001 |
| *Mean fluorescence*  *intensity of IL-1β*  *× 10^5^ (AU)* | Con vs. IFN-γ | -3.328 | One-Way ANOVA  Tukey’s post hoc tests | -4.954 to -1.701 | F (5, 18) = 140.7 | <0.0001 |
|  | IFN-γ vs. IFN-γ+fludarabin | 2.401 |  | 0.7746 to 4.028 |  | 0.0021 |
|  | LPS vs. IFN-γ+LPS | -7.081 |  | -8.708 to -5.454 |  | <0.0001 |
|  | IFN-γ+LPS vs. IFN-γ+LPS+fludarabin | 8.876 |  | 7.249 to 10.50 |  | <0.0001 |
| *IL-1β in medium*  *(pg/ml)* | Con vs. IFN-γ | -11.61 | One-Way ANOVA  Tukey’s post hoc tests | -21.95 to -1.271 | F (5, 18) = 24.45 | 0.0227 |
|  | IFN-γ vs. IFN-γ+fludarabin | 11.13 |  | 0.7934 to 21.47 |  | 0.0307 |
|  | LPS vs. IFN-γ+LPS | -11.99 |  | -22.33 to -1.649 |  | 0.0178 |
|  | IFN-γ+LPS vs. IFN-γ+LPS+fludarabin | 21.13 |  | 10.79 to 31.47 |  | <0.0001 |
| *Caspase-3^+^ cells (%)* | Con vs. IFN-γ | -8.713 | One-Way ANOVA  Tukey’s post hoc tests | -13.95 to -3.476 | F (5, 18) = 27.15 | 0.0006 |
|  | IFN-γ vs. IFN-γ+fludarabin | 8.375 |  | 3.139 to 13.61 |  | 0.0009 |
|  | LPS vs. IFN-γ+LPS | -5.988 |  | -11.22 to -0.7517 |  | 0.0198 |
|  | IFN-γ+LPS vs. IFN-γ+LPS+fludarabin | 9.627 |  | 4.391 to 14.86 |  | 0.0002 |
| *MDA*  *in medium (μM)* | Con vs. IFN-γ | -1.451 | One-Way ANOVA  Tukey’s post hoc tests | -2.037 to -0.8659 | F (5, 18) = 53.39 | <0.0001 |
|  | IFN-γ vs. IFN-γ+fludarabin | 1.281 |  | 0.6952 to 1.866 |  | <0.0001 |
|  | LPS vs. IFN-γ+LPS | -0.9879 |  | -1.573 to -0.4025 |  | 0.0005 |
|  | IFN-γ+LPS vs. IFN-γ+LPS+fludarabin | 1.257 |  | 0.6712 to 1.842 |  | <0.0001 |
| *Relative ROS*  *level in medium* | Con vs. IFN-γ | -1.203 | One-Way ANOVA  Tukey’s post hoc tests | -2.068 to -0.3376 | F (5, 18) = 19.71 | 0.0038 |
|  | IFN-γ vs. IFN-γ+fludarabin | 1.144 |  | 0.2784 to 2.009 |  | 0.0060 |
|  | LPS vs. IFN-γ+LPS | -0.9746 |  | -1.840 to -0.1095 |  | 0.0222 |
|  | IFN-γ+LPS vs. IFN-γ+LPS+fludarabin | 1.677 |  | 0.8121 to 2.542 |  | 0.0001 |
| *LDH leakage (mU/ml)* | Con vs. IFN-γ | -1.887 | One-Way ANOVA  Tukey’s post hoc tests | -2.926 to -0.8486 | F (5, 18) = 37.81 | 0.0002 |
|  | IFN-γ vs. IFN-γ+fludarabin | 1.117 |  | 0.07811 to 2.156 |  | 0.0310 |
|  | LPS vs. IFN-γ+LPS | -2.238 |  | -3.277 to -1.199 |  | <0.0001 |
|  | IFN-γ+LPS vs. IFN-γ+LPS+fludarabin | 2.477 |  | 1.439 to 3.516 |  | <0.0001 |

**Table S9．The F value and P value in multiple comparisons of figure 6**

| **Marker** | **Groups** | **Mean Diff.** | **Type of ANOVA** | **95.00% CI of diff.** | **F, DFn, Dfd** | **P value** |
| --- | --- | --- | --- | --- | --- | --- |
| *Area of Iba1^+^ staining (%)* | PBS vs. IFN-γ | -1.943 | One-Way ANOVA  Tukey’s post hoc tests | -3.507 to -0.3798 | F (6, 21) = 16.32 | 0.0090 |
|  | IFN-γ vs. IFN-γ+fludarabin | 1.842 |  | 0.2783 to 3.405 |  | 0.0144 |
|  | IFN-γ+LPS vs. IFN-γ+LPS+fludarabin | 1.877 |  | 0.3138 to 3.441 |  | 0.0122 |
| *Iba1^+^ number / mm^2^* | PBS vs. IFN-γ | -155.9 | One-Way ANOVA  Tukey’s post hoc tests | -241.0 to -70.85 | F (6, 21) = 19.39 | 0.0001 |
|  | IFN-γ vs. IFN-γ+fludarabin | 122.2 |  | 37.08 to 207.2 |  | 0.0022 |
|  | IFN-γ+LPS vs. IFN-γ+LPS+fludarabin | 128.5 |  | 43.45 to 213.6 |  | 0.0012 |
| *Area of microglial soma (μm^2^)* | PBS vs. IFN-γ | -8.998 | One-Way ANOVA  Tukey’s post hoc tests | -15.59 to -2.408 | F (6, 21) = 19.08 | 0.0036 |
|  | IFN-γ vs. IFN-γ+fludarabin | 7.495 |  | 0.9047 to 14.08 |  | 0.0193 |
|  | IFN-γ+LPS vs. IFN-γ+LPS+fludarabin | 7.377 |  | 0.7873 to 13.97 |  | 0.0218 |
| *Aggregate intersections* | PBS vs. IFN-γ | 31.83 | One-Way ANOVA  Tukey’s post hoc tests | 8.526 to 55.13 | F (6, 21) = 17.69 | 0.0036 |
|  | IFN-γ vs. IFN-γ+LPS | 28.48 |  | 5.174 to 51.78 |  | 0.0104 |
|  | IFN-γ vs. IFN-γ+fludarabin | -30.40 |  | -53.70 to -7.098 |  | 0.0057 |
|  | LPS vs. IFN-γ+LPS | 28.85 |  | 5.547 to 52.15 |  | 0.0093 |
|  | IFN-γ+LPS vs. IFN-γ+LPS+fludarabin | -29.95 |  | -53.25 to -6.647 |  | 0.0066 |
| *Length of branches (μm)* | PBS vs. IFN-γ | 40.30 | One-Way ANOVA  Tukey’s post hoc tests | 13.07 to 67.53 | F (6, 21) = 26.76 | 0.0015 |
|  | IFN-γ vs. IFN-γ+LPS | 39.08 |  | 11.85 to 66.30 |  | 0.0022 |
|  | IFN-γ vs. IFN-γ+fludarabin | -30.39 |  | -57.62 to -3.164 |  | 0.0224 |
|  | LPS vs. IFN-γ+LPS | 29.48 |  | 2.256 to 56.71 |  | 0.0283 |
|  | IFN-γ+LPS vs. IFN-γ+LPS+fludarabin | -33.03 |  | -60.25 to -5.799 |  | 0.0112 |
| *mRNA expression*  *NLRP3* | PBS vs. IFN-γ | -1.891 | One-Way ANOVA  Tukey’s post hoc tests | -3.246 to -0.5355 | F (6, 21) = 23.83 | 0.0029 |
|  | IFN-γ vs. IFN-γ+fludarabin | 1.728 |  | 0.3732 to 3.084 |  | 0.0071 |
|  | LPS vs. IFN-γ+LPS | -1.738 |  | -3.093 to -0.3827 |  | 0.0067 |
|  | IFN-γ+LPS vs. IFN-γ+LPS+fludarabin | 2.127 |  | 0.7721 to 3.483 |  | 0.0008 |
| *mRNA expression*  *Caspase-1* | PBS vs. IFN-γ | -0.8897 | One-Way ANOVA  Tukey’s post hoc tests | -1.589 to -0.1900 | F (6, 21) = 25.73 | 0.0073 |
|  | IFN-γ vs. IFN-γ+fludarabin | 1.128 |  | 0.4279 to 1.827 |  | 0.0006 |
|  | LPS vs. IFN-γ+LPS | -0.8916 |  | -1.591 to -0.1918 |  | 0.0071 |
|  | IFN-γ+LPS vs. IFN-γ+LPS+fludarabin | 1.259 |  | 0.5595 to 1.959 |  | 0.0001 |
| *mRNA expression*  *GSDMD* | PBS vs. IFN-γ | -1.254 | One-Way ANOVA  Tukey’s post hoc tests | -2.299 to -0.2096 | F (6, 21) = 24.69 | 0.0122 |
|  | IFN-γ vs. IFN-γ+fludarabin | 1.535 |  | 0.4906 to 2.580 |  | 0.0017 |
|  | LPS vs. IFN-γ+LPS | -1.307 |  | -2.351 to -0.2621 |  | 0.0085 |
|  | IFN-γ+LPS vs. IFN-γ+LPS+fludarabin | 2.396 |  | 1.352 to 3.441 |  | <0.0001 |
| *mRNA expression*  *IL-1β* | PBS vs. IFN-γ | -2.413 | One-Way ANOVA  Tukey’s post hoc tests | -4.062 to -0.7647 | F (6, 21) = 36.36 | 0.0017 |
|  | IFN-γ vs. IFN-γ+fludarabin | 2.261 |  | 0.6130 to 3.910 |  | 0.0035 |
|  | LPS vs. IFN-γ+LPS | -2.917 |  | -4.565 to -1.268 |  | 0.0002 |
|  | IFN-γ+LPS vs. IFN-γ+LPS+fludarabin | 3.086 |  | 1.438 to 4.735 |  | <0.0001 |
| *mRNA expression*  *IL-18* | PBS vs. IFN-γ | -1.162 | One-Way ANOVA  Tukey’s post hoc tests | -2.162 to -0.1613 | F (6, 21) = 30.58 | 0.0162 |
|  | IFN-γ vs. IFN-γ+fludarabin | 1.187 |  | 0.1872 to 2.188 |  | 0.0135 |
|  | LPS vs. IFN-γ+LPS | -2.138 |  | -3.139 to -1.138 |  | <0.0001 |
|  | IFN-γ+LPS vs. IFN-γ+LPS+fludarabin | 1.786 |  | 0.7856 to 2.786 |  | 0.0002 |
| *mRNA expression*  *iNOS* | PBS vs. IFN-γ | -1.609 | One-Way ANOVA  Tukey’s post hoc tests | -3.132 to -0.08569 | F (6, 21) = 35.82 | 0.0340 |
|  | IFN-γ vs. IFN-γ+fludarabin | 1.542 |  | 0.01919 to 3.065 |  | 0.0459 |
|  | LPS vs. IFN-γ+LPS | -2.580 |  | -4.103 to -1.057 |  | 0.0003 |
|  | IFN-γ+LPS vs. IFN-γ+LPS+fludarabin | 2.327 |  | 0.8043 to 3.850 |  | 0.0011 |
| *NLRP3 levels (ng/g)* | PBS vs. IFN-γ | -27.27 | One-Way ANOVA  Tukey’s post hoc tests | -48.94 to -5.598 | F (6, 21) = 13.08 | 0.0080 |
|  | IFN-γ vs. IFN-γ+fludarabin | 22.43 |  | 0.7581 to 44.10 |  | 0.0394 |
|  | LPS vs. IFN-γ+LPS | -22.69 |  | -44.36 to -1.016 |  | 0.0363 |
|  | IFN-γ+LPS vs. IFN-γ+LPS+fludarabin | 27.23 |  | 5.560 to 48.90 |  | 0.0081 |
| *IL-1β levels (pg/g)* | PBS vs. IFN-γ | -38.03 | One-Way ANOVA  Tukey’s post hoc tests | -65.12 to -10.94 | F (6, 21) = 31.63 | 0.0027 |
|  | IFN-γ vs. IFN-γ+fludarabin | 40.88 |  | 13.79 to 67.97 |  | 0.0012 |
|  | LPS vs. IFN-γ+LPS | -43.34 |  | -70.43 to -16.25 |  | 0.0006 |
|  | IFN-γ+LPS vs. IFN-γ+LPS+fludarabin | 45.12 |  | 18.03 to 72.21 |  | 0.0004 |
| *TNF-α levels (pg/g)* | PBS vs. IFN-γ | -17.73 | One-Way ANOVA  Tukey’s post hoc tests | -35.14 to -0.3166 | F (6, 21) = 17.27 | 0.0442 |
|  | IFN-γ vs. IFN-γ+fludarabin | 20.77 |  | 3.355 to 38.18 |  | 0.0129 |
|  | LPS vs. IFN-γ+LPS | -18.65 |  | -36.07 to -1.242 |  | 0.0307 |
|  | IFN-γ+LPS vs. IFN-γ+LPS+fludarabin | 19.87 |  | 2.455 to 37.28 |  | 0.0187 |
| *Relative ROS level* | PBS vs. IFN-γ | -1.033 | One-Way ANOVA  Tukey’s post hoc tests | -1.913 to -0.1530 | F (6, 21) = 21.80 | 0.0148 |
|  | IFN-γ vs. IFN-γ+fludarabin | 1.120 |  | 0.2404 to 2.000 |  | 0.0072 |
|  | LPS vs. IFN-γ+LPS | -1.166 |  | -2.046 to -0.2865 |  | 0.0049 |
|  | IFN-γ+LPS vs. IFN-γ+LPS+fludarabin | 1.009 |  | 0.1295 to 1.889 |  | 0.0179 |
| *Relative MDA levels* | PBS vs. IFN-γ | -0.9516 | One-Way ANOVA  Tukey’s post hoc tests | -1.566 to -0.3376 | F (6, 21) = 37.03 | 0.0009 |
|  | IFN-γ vs. IFN-γ+fludarabin | 1.103 |  | 0.4894 to 1.717 |  | 0.0001 |
|  | LPS vs. IFN-γ+LPS | -1.238 |  | -1.852 to -0.6238 |  | <0.0001 |
|  | IFN-γ+LPS vs. IFN-γ+LPS+fludarabin | 1.227 |  | 0.6133 to 1.841 |  | <0.0001 |
| *Relative NO levels* | PBS vs. IFN-γ | -1.433 | One-Way ANOVA  Tukey’s post hoc tests | -2.396 to -0.4701 | F (6, 21) = 35.26 | 0.0015 |
|  | IFN-γ vs. IFN-γ+fludarabin | 1.154 |  | 0.1913 to 2.117 |  | 0.0124 |
|  | LPS vs. IFN-γ+LPS | -1.112 |  | -2.074 to -0.1488 |  | 0.0170 |
|  | IFN-γ+LPS vs. IFN-γ+LPS+fludarabin | 1.456 |  | 0.4935 to 2.419 |  | 0.0012 |

**Table S10．The F value and P value in multiple comparisons of figure 7**

| **Marker** | **Groups** | **Mean Diff.** | **Type of ANOVA** | **95.00% CI of diff.** | **F, DFn, Dfd** | **P value** |
| --- | --- | --- | --- | --- | --- | --- |
| *Cleaved caspase-3^+^ cells*  *in each slice* | PBS vs. IFN-γ | -7.194 | One-Way ANOVA  Tukey’s post hoc tests | -12.62 to -1.766 | F (6, 21) = 27.45 | 0.0049 |
|  | IFN-γ vs. IFN-γ+fludarabin | 6.558 |  | 1.130 to 11.99 |  | 0.0116 |
|  | LPS vs. IFN-γ+LPS | -7.488 |  | -12.92 to -2.060 |  | 0.0033 |
|  | IFN-γ+LPS vs. IFN-γ+LPS+fludarabin | 6.627 |  | 1.200 to 12.06 |  | 0.0105 |
| *Cleaved caspase-3^+^-NeuN^+^*  *cells in each slice* | PBS vs. IFN-γ | -3.577 | One-Way ANOVA  Tukey’s post hoc tests | -5.978 to -1.175 | F (6, 21) = 25.15 | 0.0014 |
|  | IFN-γ vs. IFN-γ+fludarabin | 2.803 |  | 0.4021 to 5.205 |  | 0.0155 |
|  | LPS vs. IFN-γ+LPS | -3.856 |  | -6.257 to -1.454 |  | 0.0006 |
|  | IFN-γ+LPS vs. IFN-γ+LPS+fludarabin | 3.199 |  | 0.7979 to 5.601 |  | 0.0046 |
| *Relative mRNA levels*  *Bax* | PBS vs. IFN-γ | -2.005 | One-Way ANOVA  Tukey’s post hoc tests | -3.606 to -0.4037 | F (6, 21) = 26.28 | 0.0084 |
|  | IFN-γ vs. IFN-γ+fludarabin | 1.655 |  | 0.05368 to 3.256 |  | 0.0398 |
|  | LPS vs. IFN-γ+LPS | -2.513 |  | -4.114 to -0.9112 |  | 0.0008 |
|  | IFN-γ+LPS vs. IFN-γ+LPS+fludarabin | 3.450 |  | 1.849 to 5.051 |  | <0.0001 |
| *Relative mRNA levels*  *Bad* | PBS vs. IFN-γ | -3.330 | One-Way ANOVA  Tukey’s post hoc tests | -5.121 to -1.539 | F (6, 21) = 65.31 | <0.0001 |
|  | IFN-γ vs. IFN-γ+fludarabin | 2.853 |  | 1.061 to 4.644 |  | 0.0007 |
|  | LPS vs. IFN-γ+LPS | -2.549 |  | -4.340 to -0.7573 |  | 0.0024 |
|  | IFN-γ+LPS vs. IFN-γ+LPS+fludarabin | 5.306 |  | 3.515 to 7.098 |  | <0.0001 |
| *Traveled distances*  *in open field (m)* | PBS vs. IFN-γ+LPS | 7.376 | One-Way ANOVA  Tukey’s post hoc tests | 2.535 to 12.22 | F (6, 49) = 5.391 | 0.0004 |
|  | IFN-γ vs. IFN-γ+LPS | 5.286 |  | 0.4449 to 10.13 |  | 0.0240 |
| *Location recognition index* | PBS vs. IFN-γ | 0.1126 | One-Way ANOVA  Tukey’s post hoc tests | 0.04457 to 0.1807 | F (6, 49) = 13.71 | 0.0001 |
|  | IFN-γ vs. IFN-γ+fludarabin | 0.07740 |  | -0.1455 to -0.009335 |  | 0.0164 |
|  | LPS vs. IFN-γ+LPS | 0.07506 |  | 0.006997 to 0.1431 |  | 0.0220 |
|  | IFN-γ+LPS vs. IFN-γ+LPS+fludarabin | -0.09041 |  | -0.1585 to -0.02235 |  | 0.0029 |
| *Novel object recognition index* | PBS vs. IFN-γ | 0.09655 | One-Way ANOVA  Tukey’s post hoc tests | 0.009161 to 0.1839 | F (6, 49) = 10.81 | 0.0216 |
|  | PBS vs. IFN-γ+LPS | 0.1673 |  | 0.07994 to 0.2547 |  | <0.0001 |
|  | IFN-γ vs. IFN-γ+fludarabin | -0.1064 |  | -0.1937 to -0.01896 |  | 0.0082 |
|  | IFN-γ+LPS vs. IFN-γ+LPS+fludarabin | -0.1037 |  | -0.1911 to -0.01629 |  | 0.0107 |
| *Latency (s)* | PBS vs. IFN-γ | 13.00 | One-Way ANOVA  Tukey’s post hoc tests | 0.01099 to 25.99 | F (6, 49) = 9.502 | 0.0497 |
|  | IFN-γ vs. IFN-γ+fludarabin | -13.00 |  | -25.99 to -0.01099 |  | 0.0497 |
|  | IFN-γ+LPS vs. IFN-γ+LPS+fludarabin | -13.38 |  | -26.36 to -0.3860 |  | 0.0397 |
| *Immobility time (s)* | PBS vs. IFN-γ | -37.13 | One-Way ANOVA  Tukey’s post hoc tests | -64.06 to -10.19 | F (6, 49) = 19.80 | 0.0018 |
|  | IFN-γ vs. IFN-γ+fludarabin | 29.00 |  | 2.061 to 55.94 |  | 0.0273 |
|  | LPS vs. IFN-γ+LPS | -34.00 |  | -60.94 to -7.061 |  | 0.0054 |
|  | IFN-γ+LPS vs. IFN-γ+LPS+fludarabin | 37.00 |  | 10.06 to 63.94 |  | 0.0019 |
