# Supplementary figures and images for: Microglial priming by IFN‐γ involves STAT1‐mediated activation of the NLRP3 inflammasome
Source: CNS Neurosci Ther. 2024 Oct 11;30(10):e70061. doi: 10.1111/cns.70061 (PMC11468839; doi:10.1111/cns.70061)

Full unedited gel/blot for Figure 2I

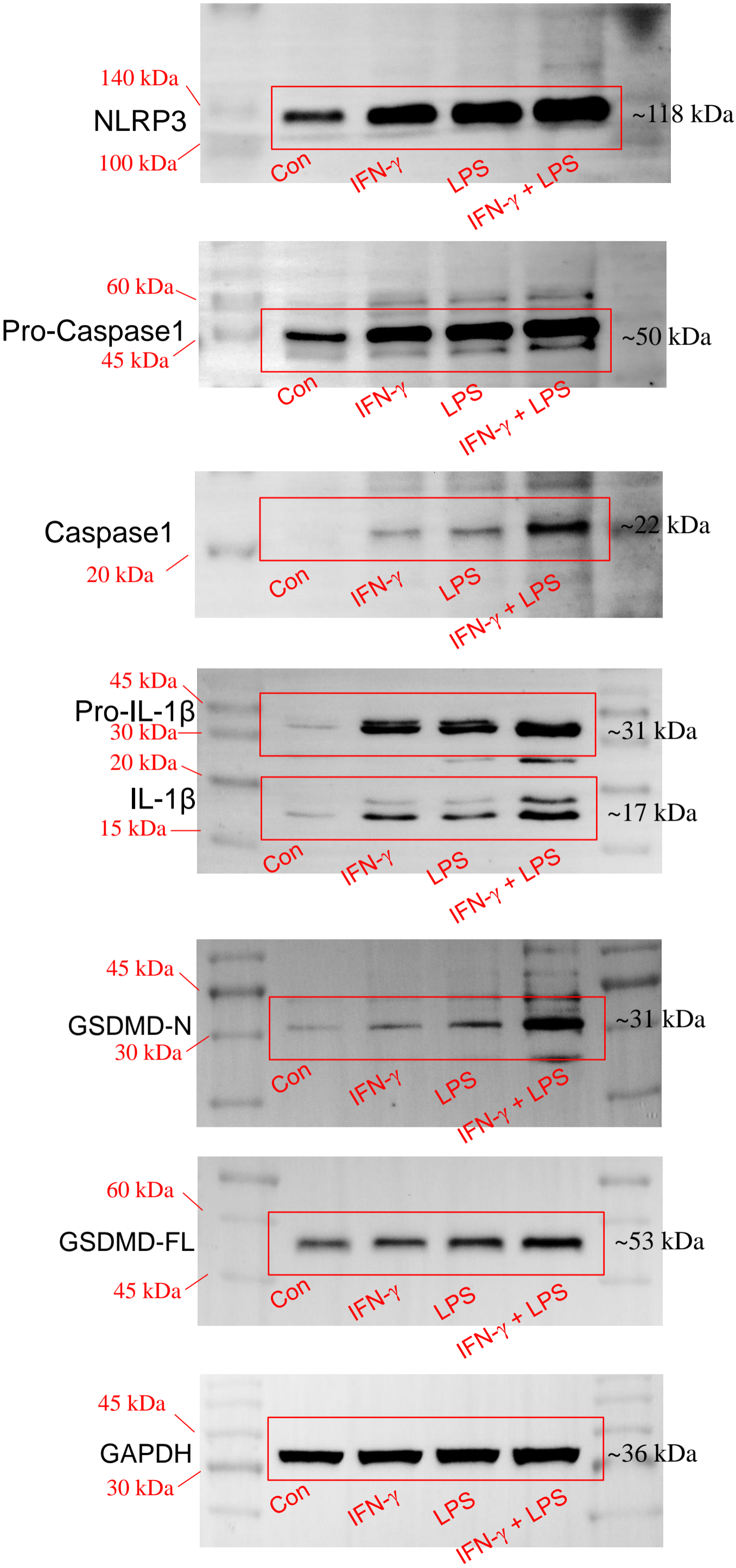

# Full unedited gel/blot for Figure 4C

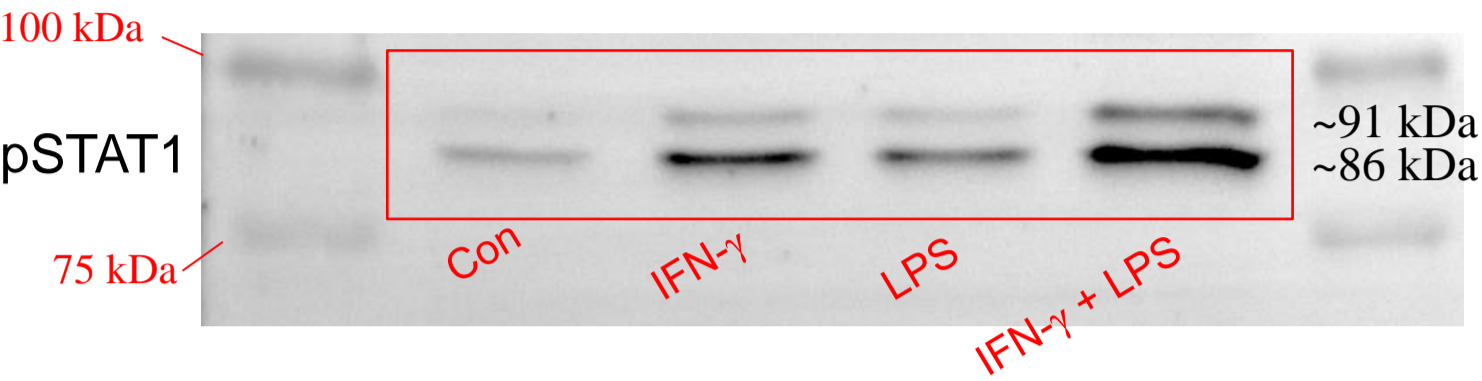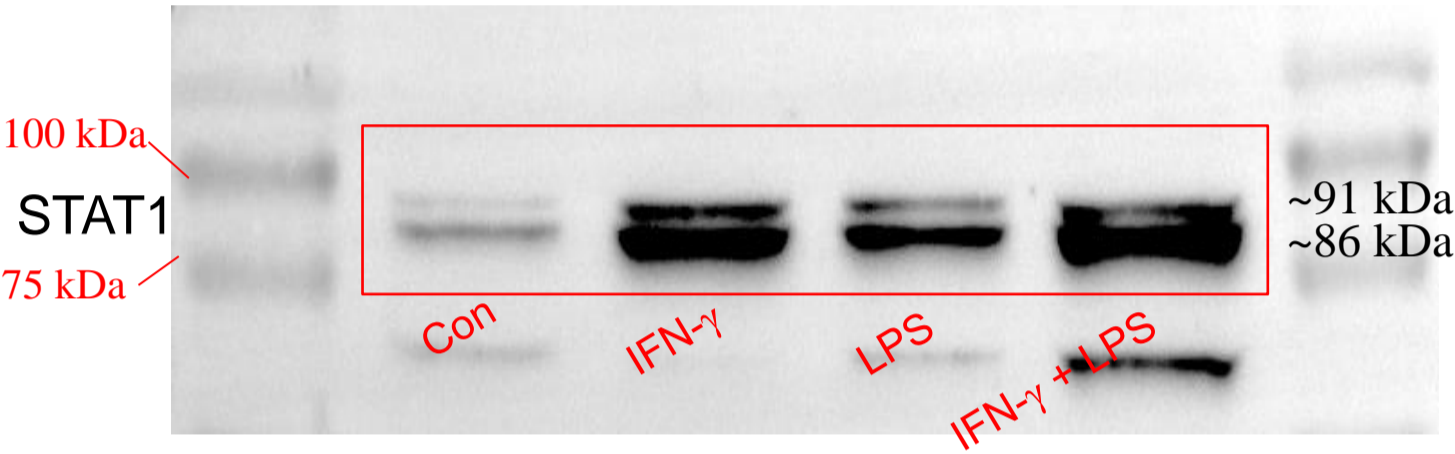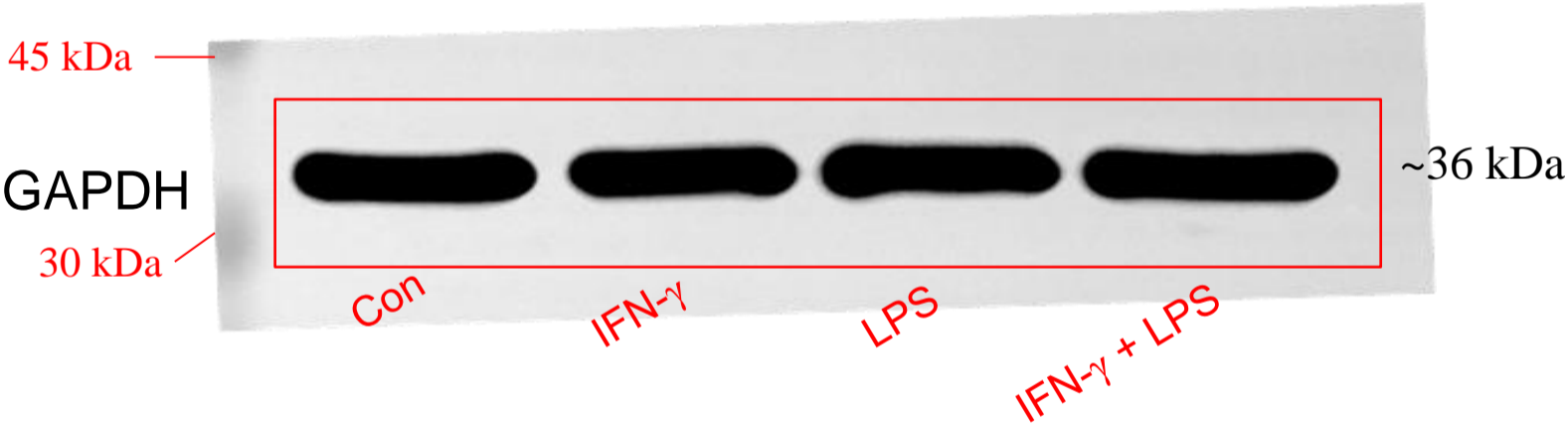

Supplement: Supplementary file 1 — Data S1. [file CNS-30-e70061-s001.zip › Full unedited gel.pdf]
